# Regulation of CCL2 by EZH2 affects tumor-associated macrophages polarization and infiltration in breast cancer

**DOI:** 10.1038/s41419-022-05169-x

**Published:** 2022-08-29

**Authors:** Ya-fang Wang, Lei Yu, Zong-long Hu, Yan-fen Fang, Yan-yan Shen, Min-fang Song, Yi Chen

**Affiliations:** 1 Division of Anti-Tumor Pharmacology, Shanghai Institute of Materia Medica, Chinese Academy of Sciences, Shanghai, PR China; 2 Shanghai Institute for Advanced Immunochemical Studies, ShanghaiTech University, Shanghai, PR China; 3 University of Chinese Academy of Sciences, Beijing, China; 4 School of Life Science and Technology, ShanghaiTech University, Shanghai, PR China

**Keywords:** Cancer microenvironment, Target validation, Breast cancer

## Abstract

Tumor associated macrophages (TAMs) play an important role in tumorigenesis, development and anti-cancer drug therapy. However, very few epigenetic compounds have been elucidated to affect tumor growth by educating TAMs in the tumor microenvironment (TME). Herein, we identified that EZH2 performs a crucial role in the regulation of TAMs infiltration and protumoral polarization by interacting with human breast cancer (BC) cells. We showed that EZH2 inhibitors-treated BC cells induced M2 macrophage polarization in vitro and in vivo, while EZH2 knockdown exhibited the opposite effect. Mechanistically, inhibition of EZH2 histone methyltransferase alone by EZH2 inhibitors in breast cancer cells could reduce the enrichment of H3K27me3 on *CCL2* gene promoter, elevate CCL2 transcription and secretion, contributing to the induction of M2 macrophage polarization and recruitment in TME, which reveal a potential explanation behind the frustrating results of EZH2 inhibitors against breast cancer. On the contrary, EZH2 depletion led to DNA demethylation and subsequent upregulation of miR-124-3p level, which inhibited its target CCL2 expression in the tumor cells, causing arrest of TAMs M2 polarization. Taken together, these data suggested that EZH2 can exert opposite regulatory effects on TAMs polarization through its enzymatic or non-enzymatic activities. Our results also imply that the effect of antitumor drugs on TAMs may affect its therapeutic efficacy, and the combined application with TAMs modifiers should be warranted to achieve great clinical success.

## Introduction

The tumor microenvironment (TME) is composed of tumor cells, immune cells, stromal cells, cytokines and chemokines. Under the influence of various genetic and environmental factors, TME undergoes dynamic transformation and intercellular interaction, which plays a crucial role in the tumor development [1]. It has been well known that tumor-associated macrophages (TAMs) are recognized as a major component of TME, and its state determines whether to promote tumor progression or not [2]. Usually, macrophages are classified as two major subsets: classical activation M1-type and alternative activation M2-type [3]. It has been revealed that M1 TAMs restrain cancer development, whereas M2 TAMs often play a pro-tumorigenic role in which they facilitate tumor cells migration and metastasis by enhancing immunosuppression, angiogenesis, and extracellular matrix remodeling [4]. Infiltration or enrichment of M2 TAMs in tumors is associated with a poor prognosis in many human tumors, especially when these cells are observed at the invasive front of the primary breast tumors [5]. In addition, the phenotype of TAMs in the tumors can be changed in response to the TME. Various cytokines secreted by tumor cells including breast cancer (BC) cells have been demonstrated to modulate M2 polarization and infiltration in TME, such as colony-stimulating factor 1 (CSF1) [6], interleukin10 (IL-10) [7], chemokine (C-C motif) ligand 2 (CCL2, also known as MCP-1) [8], transforming growth factor beta (TGF-β) [9], and etc. It’s also well recognized that the acquired drug resistance of existing anti-cancer therapy may be mediated by their effects on TAMs [10]. Therefore, inhibiting protumoral macrophages or their suppressive effects and transforming TAMs into an antitumor phenotype reveal new opportunities for anti-cancer therapy.

Enhancer of zeste homolog 2 (EZH2) is the catalytic core subunit of Polycomb Repressive Complex 2 (PRC2) that can silence target genes through trimethylation of H3K27 [11]. Notably, high level of EZH2 is implicated in tumorigenesis and correlates with poor prognosis in various tumor types, including breast cancer, ovarian cancer, etc. [12, 13]. Emerging researches suggest that EZH2 functions as a multifaceted molecule which exerts its canonical role in transcriptional silencing as a histone methyltransferase or activates genes that promote oncogenesis, tumor growth and metastasis independent of PRC2 [14, 15]. It plays a key role in regulating DNA repair and genomic stability in numerous malignant tumor models. EZH2 also has been shown to regulate inflammatory cytokines and chemokines in several different cell types including cancer cells [16, 17], CD4^+^ T cells [18], regulatory T cells [19], Kupffer cells [20], and macrophages [21]. Studies have revealed that EZH2 elevates hepatocyte growth factor (HGF) and macrophage migration inhibitory factor (MIF) in hepatocellular carcinoma, which contribute to M2 repolarization and poor prognosis [22]. All these make EZH2 as an important anticancer target in the current epigenetic strategies [23, 24]. And a series of EZH2 inhibitors, including tazemetostat (EPZ-6438), E11, GSK126 etc., have demonstrated antitumor effects in preclinical and clinical models [25]. Especially, EPZ-6438 received accelerated approval in 2020 in the USA for the treatment of advanced epithelioid sarcoma and certain types of follicular lymphoma [26]. However, increasing evidence suggests a limited antitumor effect of EZH2 inhibitors in preclinical and clinical studies. Just as EZH2 has been recognized as a marker of aggressive breast cancer and is associated with poor survival [27, 28], downregulation of EZH2 decreases the growth and metastasis of invasive breast carcinoma [29, 30], but EZH2 inhibitors alone have shown little benefit against breast cancer [31, 32]. And till now, the precise mechanism by which EZH2 affects TAMs polarization and breast cancer progression remains unclear. Therefore, we focused on exploring the role of EZH2 inhibitors in TAMs differentiation in this study, which may influence the efficacy of EZH2 inhibitors against breast cancer. We found that breast tumor cells treated with EZH2 small-molecule inhibitors altered the secretome of inflammatory mediators, thus stimulating TAMs infiltration and polarization to a tumor-promoting phenotype, whereas EZH2 genetical deletion rendered the opposite effect. Further, we attempted to figure out the specific regulatory mechanism, which might be one of the reasons for limiting the benefits of EZH2 inhibitors against breast cancer.

## Materials and methods

### Cell lines and reagents

Human breast cancer cell lines (MDA-MB-231, MCF7, BT549), mouse breast cancer cell line 4T1, and human acute monocyte leukemia cell line (THP-1) and human embryonic kidney 293T were purchased from the American Type Culture Collection (ATCC, Manassas, VA, USA). The above cell lines were verified by short tandem repeat sequence identification of the cell bank, and all of them were free of mycoplasma contamination. Cells were cultured and maintained according to routine procedures. MCF7 were maintained in Eagle’s Minimum Essential Medium (MEM, Invitrogen, Shanghai, China). BT549, THP-1 and 4T1 were kept in RPMI-1640 Medium. 293T cells were kept in Dulbecco’s Modified Eagle’s Medium (DMEM). And these cell lines were cultured in a suitable incubator with a humidified atmosphere of 5% CO_2_ at 37 °C. MDA-MB-231 cells were cultured in Leibovitz’s L-15 Medium at atmospheric air and 37 °C. The complete growth media were supplemented with 10% fetal bovine serum (FBS), 100 U/ml penicillin and 100 mg/ml streptomycin.

EPZ-6438, Gefitinib were both purchased from Selleck (Shanghai, China). Bindarit (CCL2 inhibitor), RS 504393 (CCR2 antagonist) and GSK126 were purchased from MedChemExpress (Monmouth Junction, NJ, USA). BMS CCR2 22 (CCR2 Antagonist) was purchased from Tocris Bioscience (Ellisville, MO). Phorbol 12-myristate 13-acetate (PMA), Lipopolysaccharides (LPS) were bought from Sigma Aldrich (St. Louis, MO, USA). Specific neutralizing antibodies against CCL2 or IL-10 were purchased from R&D Systems. Murine and human recombinant proteins including M-CSF, IFNγ, IL-4, IL-13, CCL2, IL-10 were purchased from PeproTech Inc. The other key resources are listed in Table S1.

### Generation of M2 macrophages

Human monocyte THP-1 cells were differentiated and polarized according to established protocols [20, 27], with minor modifications. Briefly, THP-1 cells were incubated with 320 nM PMA for 6 hour (h), and then 20 ng/ml IL-4 and 20 ng/ml IL-13 were added for another 66 h of a total incubation time of 72 h to generate M2-polarized macrophages.

Bone marrow-derived macrophages (BMDM)-derived M2 macrophage were isolated from bone marrow of BALB/c mice. Briefly, after lysis of red blood cells, BMDM were cultured in DMEM media containing 10% FBS and 50 ng/ml M-CSF (R&D Systems) for 6 days followed by IL-4 (10 ng/mL) and IL-13 (10 ng/mL) stimulation for one day more to establish M2 macrophages. The animal protocol was approved by Institute Animal Care and Use Committee at Shanghai Institute of Materia Medica.

### Cell proliferation and in vitro migration assay

To analyze cell proliferation, 2–4 × 10^3^ cells were seeded onto a 96-well culture plate with 100 μL conditioned medium (CM) and collected for Sulforhodamine B (SRB) assay after indicated time periods. The cells were fixed with 10% trichloroacetic acid (TCA), washed with distilled water, and stained with sulforhodamine B (SRB; Sigma) in 1% acetic acid. SRB left on the cells was dissolved using 10 mM Tris-HCl, and optical density was measured at 560 nm by using a microplate reader (Bio-Rad).

To perform transwell migration assay, plates with permeable polycarbonate membrane inserts (8 μm pore-size, Corning, NY, USA) were used. The upper and lower chambers were plated with BMDM or THP-1 cells and 4T1 or MDA-MB-231 cells respectively. CCL2 neutralizing antibody and control antibody were added into both sides of the insert at 5 μg/mL. After 12–24 h, plates were fixed with 4% (w/v) paraformaldehyde and cell migration was assessed based on the number of cells that migrated through the insert by crystal violet staining (3 different randomly selected 10× fields in each insert).

### RNA extraction and RT-qPCR analysis

Primary BMDM, THP-1 cells or BC cells were seeded and treated as indicated for 48 h. The cells were harvested with Trizol (Invitrogen, Carlsbad, CA, USA) and mRNAs were extracted according to the procedure. Quantitative Real-time PCR (RT-qPCR) was done with a Perkin-Elmer GeneAmp PCR System 7500 (Waltham, MA, USA). The PCR primers are listed in Supplementary Table S3. To obtain the relative gene expressions, the calculated quantity of the target genes for each sample were divided by the average sample quantity of the housekeeping gene RPS18 (mouse) or GAPDH (human) using the ΔΔCT method.

### Western blotting and enzyme linked immunosorbent assay (ELISA)

After treatments, cells were harvested and lysed using RIPA lysis buffer (Beyotime, Biotechnology, China) containing protease inhibitor cocktail (Roche). Protein concentrations were detected using the BCA method, and equal amounts of protein for each lysate sample were subject to SDS-PAGE at 80–130 V for 90–120 min, and then transferred to nitrocellulose filter (NC) membrane (Millipore, Boston, MA, USA). After blockade in tris-buffered saline and Tween-20 (TBS-T; 10 mM Tris, 150 mM NaCl, and 0.1% Tween-20) containing 5% nonfat dry milk or 3% bovine serum albumin (BSA), the membranes were incubated with indicated primary antibodies diluted in BSA buffer, overnight at 4 °C. The membranes were washed three times in TBS-T followed by 1 h incubation with secondary antibodies conjugated with horseradish peroxidase (HRP) at room temperature. Then the membranes were washed before enhanced chemiluminescence exposure. GAPDH or β-actin were used as housekeeping controls. Chemiluminescent detection was carried out on the ChemiDoc™ XRS + System with Image Lab™ Software (Bio-Rad, Hercules, USA). Antibody information was showed in Table S1.

For cytokine concentration measurements, ELISA was performed according to manufacturer’s instructions using cell supernatants following incubation and centrifugation. The ELISA kits are listed in Table S1.

### Plasmids’ construction and transfection

The guide RNA of EZH2 fused with CRISPR-cas9 protein was from plasmid pLenti-sgEZH2 E9.3 (addgene plasmid #90684) and the pLenti-sgRNA (addgene plasmid #71409) was used as negative control. The LV3-shCCL2 viruses were designed and constructed by GenePharma (Shanghai, China). Transfected cells were selected in complete medium containing 0.5 μg/mL puromycin (ThermoFisher Scientific, #A1113802) for 2–3 weeks. Individual subclones were isolated in 96-well plates and EZH2 knockdown clones screened by western blotting and genotyped by PCR to verify exon insertion of the Puro/RFP cassette. The puromycin concentration was reduced to 0.2 μg/mL for stable cell maintenance.

For siRNA and miRNA transient transfection, breast cancer cells were seeded into six-well plates at about 50% confluence and transfected with specific siRNAs duplex using Lipofectamine RNAiMAX Reagent Agent (Life Technologies, #13778-150) according to the manufacturer’s instructions for 48 h. Has/Mmu-miR-124-3p mimic and mimic negative control (NC), has/mmu-miR-124-3p inhibitor, and inhibitor negative control (NC) were designed and purchased from RiboBio (Guangzhou, China). EZH2 siRNAs were purchased from GenePharma (Shanghai, China). The sequences of the siRNAs used in this study are shown in Table S2.

### Three-dimensional macrophage infiltration assay

The tumor cells and macrophages were harvested, counted and prepared as a single cell suspension. Green GFP-fluorescent cancer cells 4T1 were re-suspended in 100 μL 1× Spheroid Formation ECM and seeded into to the 3D culture qualified 96 well spheroid formation plate with a final concentration of 20,000 cancer cells per well. The plates were centrifuged at 200 × *g* for 5 min at room temperature in a swinging bucket rotor and incubated at 37 °C for 1 to 3 days to promote spheroid formation. Then 100 µL of invasion matrix plus red mCherry-fluorescent BMDM cells (± 5000 macrophage cells) per well were added and plates were centrifuged at 300 × *g* at 4 °C for 5 min in a swinging bucket rotor to eliminate bubbles and position spheroids within the invasion matrix towards the middle of the well. The spheroid in each well was photographed every 24 h using the 4× objective by Fluorescence Inversion Microscope System (Leica DMIRB). Five to six days later at the end of the experiment, the fluorescent images of the 3D spheroids were obtained on a laser scanning confocal microscope (Zeiss LSM 800). Representative pictures were recorded using a Z-stack. The number of infiltrated macrophages for each sample was presented as accumulative average intensity of fluorescence using ZEN lite software.

### Transwell co-cultures

For co-cultures with M0 or M2 macrophages, pre-treated 2 × 10^5^ 4T1, MDA-MB-231 cells were seeded in 1 mL DMEM containing 10% FBS into the upper transwell chamber (0.4 μm pore size; Corning) and allowed to adhere overnight. The next day, 2 × 10^6^ macrophages in 2 mL DMEM containing 0.5% fetal bovine serum were seeded into the lower well and co-cultured for 24 h. Then the macrophages were collected for RT-PCR or western-blot analysis. Transwell co-cultures of 4T1/BMDM and MDA-MB-231/THP-1 cells were performed as described by Stewart and colleagues [33, 34].

### Tissue immunofluorescence

To detect the infiltration of TAMs in vivo, 4-week-old male BALB/c nude mice were obtained from the Shanghai Laboratory Animal Center and used in our experiments. A total of 5 × 10^6^ MDA-MB-231 cells (shcon or shCCL2 cells) were inoculated into the right flank of mice subcutaneously. EPZ-6438 was given by oral gavage 200 mg/kg/day in the treatment group as indicated. Nearly four weeks later, tumor tissues harvested from mice were frozen in Tissue-Tek O.C.T. compound and sectioned in Leica CM3050 S Cryostats, fixed in acetone, washed and blocked with PBS containing 5% goat serum. The immunodetection of F4/80 and CD206 were carried out by incubating cells with rabbit anti-CD206 and mouse anti-F4/80 primary antibody diluted 1:200 in PBS containing 3% BSA. After incubation overnight, the slides were washed three times with PBS and incubated for 1 h at room temperature with Alexa Fluor 488-conjugate goat anti-rabbit IgG (H + L) and Alexa Fluor 633-conjugate goat anti-mouse IgG (H + L) secondary antibody (ThermoFisher Scientific, USA) diluted 1:200 in PBS containing 3% BSA. Nuclei were stained using 4ʹ,6-diamidino-2-phenylindole (DAPI). The fluorescence signals were analyzed using an Olympus Fluorview 1000 confocal microscope.

### Flow cytometry analysis

Macrophages were detected by flow cytometry as described previously. Briefly, fresh tumor tissue from mice (*n* = 6/per group) were cut into pieces and digested with collagenase IV and DNase I solution (1 mg/mL) in a centrifuge tube containing 5 mL of cell culture medium, and then teared apart into single-cell suspension and passed through a 70 μm cell strainer to exclude the debris and centrifuged at 1500 × *g* for 8 min at 4 °C. The red blood cells were lysed using 1 mL RBC Lysis Buffer. Then the cells were incubated with conjugated monoclonal antibodies APC-Cy7 anti-mouse-F4/80 (1:100, Clone BM8, Cat#123107, Biolegend), PE anti-mouse-CD206 (1:100, Clone C068C2, Cat# 141706, Biolegend) or PE anti-mouse CD86 (1:100, Clone GL-1, Cat# 105008, Biolegend) diluted in FACS buffer at room temperature for 30 min. After washed twice, the samples were analyzed on a four-laser Becton-Dickinson FACS Calibur (BD Biosciences) and the data was analyzed by Flow Jo software.

### miRNA microarray analysis

Total miRNA was isolated from MDA-MB-231 cells treated with EPZ-6438 or siEZH2 using a miRNeasy Mini kit (Qiagen, Inc.) and analyzed on an Agilent 2100 Bioanalyzer (Agilent Technologies, Inc.) according to the manufacturer’s protocol. Significance between normalized expressions from array samples was determined using Student’s t-test and *p* < 0.02. Data from the microarray experiments were collected and analyzed in accordance with the MIAME guidelines. MicroRNA expression profiles were analyzed using IPA software 9 core analysis function and microRNA target filter. miRNA target prediction was performed using TargetScan 7.2 and PicTar. Unless otherwise noted, the data represent the average of the three experiments, and the error bar represents the standard error of the average.

### DNA isolation and bisulfite conversion

Cell genomic DNA was extracted using DNA Purification Kit (Qiagen, USA) according to the protocols followed by quality measurements. Unmethylated cytosine residues were converted to uracil by bisulfite treatment of 500 ng DNA using EpiTect Bisulfite Kit (Qiagen) and QiaCube automated purification system (Qiagen) according to the manufacturer’s procedures. After conversion, the final concentration of DNA was 12.5 ng/μL.

### Methylation-specific PCR (MSP)

Genomic DNA from cells and tissues was extracted with the DNeasy Tissue Kit (Qiagen Inc., Valencia, CA) according to the manufacturer’s instructions. After genomic DNA quantification, 1 mg of genomic DNA underwent bisulfite modification utilizing the EpiTect Bisulfite Kit (Qiagen). The bisulfite-converted DNA was amplified in a total volume of 20 μL PCR reaction solution. To detect the methylation status of CpG sites, primers were designed using the MethPrimer program (http://itsa.ucsf.edu/,urolab/methprimer). Primer sequences for unmethylated PCR (MSP-U) and methylatedreaction (MSP-M) are listed as in Table S3. Final products were electrophoresed for 20 min at 130 V in a 1% agarose gel. A single band in unmethylated PCR product indicated both alleles of the gene were unmethylated. The presence of a product only in the methylated reaction indicated both alleles of the gene were methylated. Samples with partially methylated alleles were positive for both methylated and unmethylated reactions.

### Cytokine antibody arrays

Human cytokine antibody array kit (AAH-CYT-G3) and human cytokine antibody array kit (GSH-CAA-440) were purchased from RayBiotech. Cells were cultured in serum-free media for 24 h after indicated treatments. Supernatants were collected and centrifuged at 10,000 g for 10 min to remove cell debris. Cytokine array membranes were blocked with blocking buffer for 1 h at room temperature. Supernatants were incubated with these membranes for 3 h. After washing with a washing buffer, membranes were incubated with primary antibody for 3 h. After washing step again, membranes were incubated with second antibody. Membrane-bound proteins were detected using a laser scanner (Innopsys’ InnoScan) using cy3 (excitation frequency = 532 nm). Relative bands intensities were determined by quantitation of each band with RayBiotech analysis tool software.

### Animal studies

All animal experiments were performed according to the institutional ethical guidelines on animal care and were approved by the Institute Animal Care and Use Committee at Shanghai Institute of Materia Medica (No. 2017-04-DJ-26, No.2019-05-DJ-47, No.2019-05-DJ-48). Random animals were assigned to each treatment group. Cancer cells or patient tissue fragments were implanted subcutaneously on the right axilla of BALB/c nude or SCID female mice (4 to 8 weeks old) provided by Shanghai Laboratory Animal Center. Tumor volumes (V) were calculated using the formula: V = ½ × length × width^2^ and measured two times per week (*n* = 6 mice for each group). In the lung metastasis study, 1 × 10^5^ 4T1 cells (4T1 shcon or 4T1 shCCL2 cells) were injected into the tail veins of 4-week-old athymic mice (*n* = 10 mice for each group). The lungs were fixed with 10% formalin after treatment and the numbers of metastatic nodules were counted. Then lung tissues were subjected to hematoxylin and eosin (H&E) staining and immunohistochemistry (IHC) examination. EPZ-6438 was given by oral gavage 200 mg/kg/day in the treatment group as indicated. Investigators were blinded to the group allocation when assessing the results.

### Patient tumor tissue samples and immunohistochemical (IHC) staining

Studies on human tissue specimens were approved and conducted by Xinchao Company. Human breast cancer (*n* = 139) tissue specimens were obtained from patients undergoing surgical resection as primary treatment at Fudan University Cancer Center (Shanghai, China) between 2005 and 2014, and written informed consents in all cases were obtained from patients at the enrollment time. IHC staining was carried out using a modified ABC method. Briefly, patient tumor samples were deparaffinized and rehydrated. Antigen retrieval was carried out by heating in 0.01 M sodium citrate buffer (pH 6.0) using a microwave oven. Then the sections were treated with 1% hydrogen peroxide in methanol for 30 min to block endogenous peroxidase activity. After 1 h preincubation in 10% normal serum to prevent nonspecific staining, the samples were incubated with primary antibodies specific to EZH2 and CD163 (1:200) at 4 °C overnight. The sections were then incubated with biotinylated secondary antibody, followed by treatment with avidin-biotin peroxidase complex solution for 1 h at room temperature. Color was developed with the 3-amino-9-ethylcarbazole (AEC) solution. Counterstaining was carried out using Mayer’s hematoxylin staining solution. Immunostaining positivity was assessed semi-quantitatively using staining intensity and percentage by two independent pathological investigators. H score was determined by multiplying the staining intensity by the percentage of positive tumor cells, with 0 representing low or undetectable staining, 1 representing intermediate staining, and 2 representing intense staining. The data were analyzed by Pearson’s χ^2^-test using statistical software SPSS version 21.0. A *p* value < 0.05 was defined as statistically significant.

### Statistical analyses

The significance of miR-124-3p expression in breast cancer patients 10-year overall survival (OS) was calculated by the Kaplan-Meier method. Correlation was studied using the Spearman test. All other data were analyzed with one-way or one-way RM analysis of variance (ANOVA) or a paired or an unpaired two-tailed t test. All experiments were repeated at least three times. The data were plotted as the mean ± SD (or SEM) using GraphPad Prism software. The threshold for statistical significance was set to *p* < 0.05.

## Results

### EZH2 inhibitors or knockdown differently affect TAMs infiltration in vivo

**Fig. 1:**
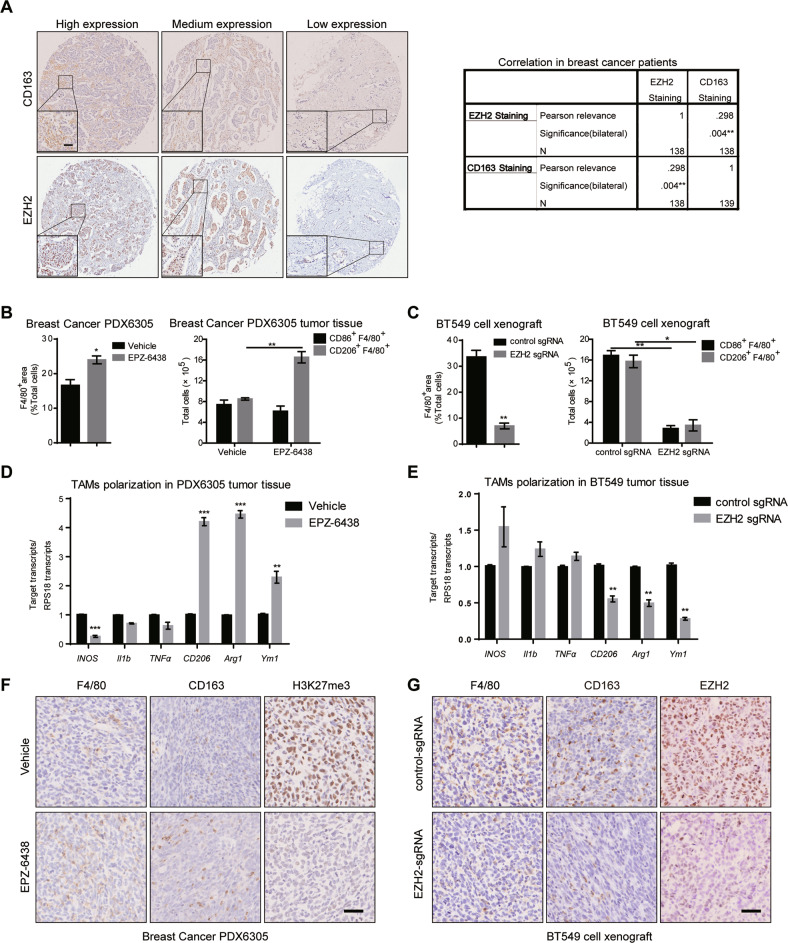
EZH2 depletion and inhibition differently affects BC cell growth and TAMs polarization. **A** Expression and the correlation of CD163 and EZH2 were examined by IHC in breast cancer patients from a tissue microarray (*n* = 139). Scale bar, 50 μm. The percentage of total macrophages, M1 and M2 macrophages in 200 mg/kg/day EPZ-6438-treated PDX6305 tumor tissues **B** and BT549 EZH2-knockdown tumor tissues **C** were analyzed by flow cytometry. RT-qPCR was applied to detect the expressions of M1 markers (*iNOS, Il1b, TNFα*) and M2 markers (*CD206, Arg1, Ym1*) in EPZ-6438-treated PDX6305 tumor tissues **D** or BT549 EZH2-knockdown tumor tissues **E**. **F** Immunohistochemistry of F4/80, CD163 and H3K27me3 in tumor tissues treated with or without EPZ-6438. **G** Immunohistochemistry of F4/80, CD163 and EZH2 in mock and EZH2 knockdown tumor tissues. Scale bar, 50 μm. In **B**–**E**, all data were shown as mean values ± SD (*n* = 3). Statistical significance was addressed using unpaired, two‐tailed Student’s t‐test, *p < 0.05, **p < 0.01, ***p < 0.001, NS, not significant. The statistical significance was calculated compared to the negative control (NC) in each group. The shown were representative of replicates and the experiments were repeated three times.

It has been proved that high level of EZH2 is found in metastatic breast cancer and confers a significantly poor prognosis. EZH2 depletion suppresses the growth and invasion of BC cells while EZH2 inhibitors alone exert little anti-tumor effect [35, 36]. And tons of literature have reported the important role of TAMs, whose polarization state and accumulation significantly correlate with tumor progression [34, 35], so we tried to explore the relationship between EZH2 expression and TAMs polarization in clinical BC patients at first. To visually distinguish the phenotype of M2 macrophages, the expression of CD163, a marker of M2 TAMs, was examined by immunohistochemistry (IHC) in a commercial human breast cancer tissue array containing 139 tumor sections. A higher density of CD163^+^ TAMs was found in tumor tissues with stronger EZH2 staining, showing a significant positive correlation (Fig. 1A). This finding suggested that EZH2 inhibitors might inhibit M2 macrophages polarization and infiltration to impair breast cancer progression. Then the effect of EZH2 inhibitors on TAMs polarization and infiltration was assessed in a breast cancer patient-derived xenograft (PDX) model (BC-PDX6305). As protumoral and proangiogenic macrophages, it is well known that M2 TAMs express elevated levels of mannose receptor-1 (CD206), arginase-1 (Arg1), Ym-1. Conversely, antitumoral and proinflammatory M1 TAMs express higher levels of markers such as inducible nitric oxide synthase (INOS), interleukin-1β (IL-1β) and tumor necrosis factor-alpha (TNFα) [37]. Mice bearing tumors were treated with 200 mg/kg EZH2 inhibitor EPZ-6438 per day, a dose that exhibited dramatic tumor growth inhibition in the preclinical lymphoma xenograft models, and then xenografts were collected [38]. EPZ-6438 showed little anti-tumor effect on this breast cancer PDX model (Fig. S1A), just like many other solid tumors that have been reported [32]. To our surprise, by flow cytometry analysis, the M2 TAMs (CD206^+^F4/80^+^) were increased by nearly two-fold in EPZ-6438-treated BC-PDX6305 tumor tissues (Fig. 1B), meanwhile the number of M1 TAMs (CD86^+^F4/80^+^) decreased a little bit. We further confirmed the results by quantitative real-time PCR (RT-qPCR) and IHC. We found that M2 marker genes were significantly highly expressed in EZH2 inhibitor-treated tumor tissues (Fig. 1D), while M1 markers didn’t show any consistent changes. And in IHC assay, compared with the vehicle group, more CD163^+^F4/80^+^ cells infiltrated in EZH2 inhibitor-treated BC tumors (Fig. 1F). These dada were inconsistent with our clinical tissue array, so the effect of EZH2 knockdown on TAMs was further assessed. BT549 cells were stably infected with CRISPR/cas9-mediated control sgRNA or an effective EZH2 sgRNA, and the knockdown efficiency was confirmed by western blot and RT-qPCR (Fig. S1B, C). According to the flow cytometry and IHC analysis, both infiltrated M1 (CD86^+^F4/80^+^) and M2 (CD206^+^F4/80^+^) TAMs were reduced in EZH2-depleted BT549 tumors (Fig. 1C, G and S1D). And all tested M2 marker genes such as CD206, Arg1 and Ym-1 were dramatically decreased in EZH2 silencing tumors (Fig. 1E). These results demonstrated that EZH2 overexpression shifts TAMs towards the M2 phenotype in vivo, leading to remodeling of the TME of breast cancer.

### EZH2 inhibitor-treated BC cells induces M2 macrophage polarization and infiltration

**Fig. 2:**
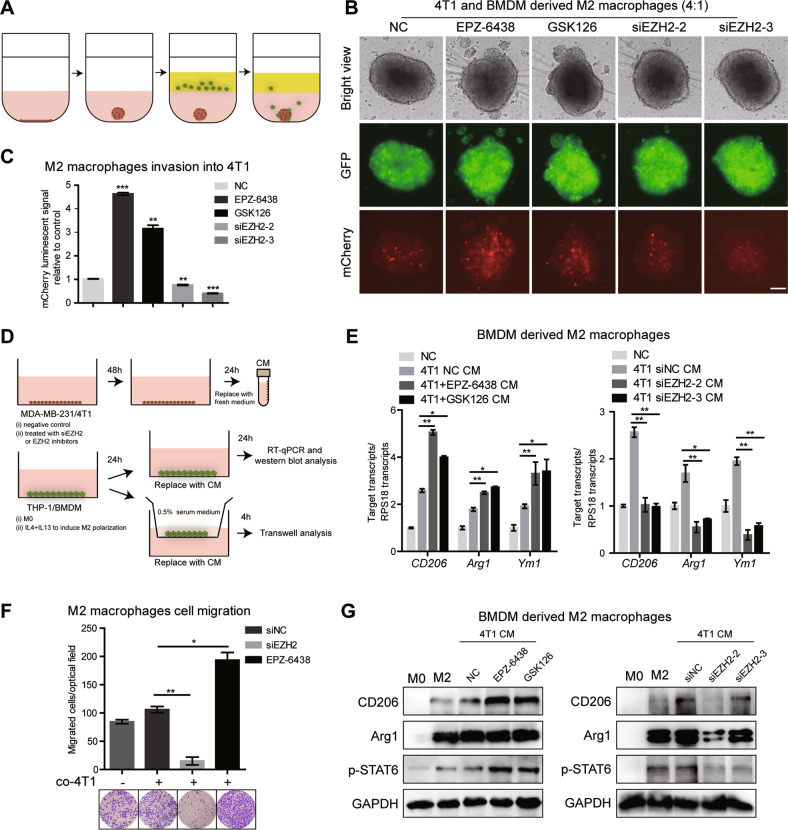
EZH2 inhibitor-treated BC cells activate TAMs to a M2 phenotype. **A** Scheme of the workflow. 4T1 cells labeled with GFP, a green tracking dye were transiently transfected with siEZH2 or treated with EPZ-6438, and seeded in a 3D culture qualified 96 well spheroid formation plate for spheroid formation. After 3-6 days, spheroids were formed and 100 µl of invasion matrix containing BMDM cells polarized by IL-4 and IL-13 treatment and labeled with mCherry, a red tracking dye were added. The cell number ratio of 4T1 and BMDM was close to 4:1. Representative photographs of infiltrating macrophages into the 4T1 microsphere (magnification, ×20) were shown **B** and relative mCherry luminescence signals were quantified **C**. Scale bar represents 100 μm. **D** Scheme of the workflow. BC cells were transiently transfected with siEZH2 or treated with EPZ-6438. CM was used as a chemoattractant for macrophages in a transwell migration assay. **E** The relative transcript levels of the M2-related genes in the CM-treated BMDM were determined by RT-qPCR. **F** The migration rates of BMDM-derived M2 macrophages with or without the co-culture of 4T1 CM were determined by transwell assay. **G** The expression of M2 macrophage markers in co-cultured BMDMs were tested by western blot. The graphs were shown as means ± SD, *n* = 3 independent experiments; *p < 0.05; **p < 0.01; ***p < 0.001, compared with control, p values were obtained using a two-tailed Student’s t test.

We next investigated the role of EZH2 inhibitor or knockdown on TAMs polarization in vitro. An in vitro 3D co-culture invasion assay was established to investigate the effect of pharmacological treated tumor cells on macrophage infiltration behavior. GFP labeled mouse BC 4T1 cells with different treatments were seeded into the extracellular matrix medium (ECM) and formed tumor spheroids alone. BMDM cells were differentiated to M2 phenotype by M-CSF and following IL-4 and IL-13. Then, these M2 macrophages were labeled with mCherry, a red cell-tracking dye, added into the invasion matrix medium and cultured with tumor spheroids for another 5–6 days (Fig. 2A). Quantification of 3D reconstructed confocal images showed that compared with the control, the number of macrophages directly infiltrating into 4T1 spheroids was remarkably increased after treatment of EZH2 inhibitor EPZ-6438 or GSK126. Consistent with in vivo data, loss of EZH2 in 4T1 still formed spheroids, but led to significant inhibition of macrophage infiltration in spheroids (Fig. 2B, C). Further, we used a cell-based assay, in which naïve or M2-polarized murine BMDMs were exposed to the conditioned medium (CM) derived from tumor cells to mimic the microenvironment interactions (Fig. 2D). As expected, CM from 4T1 cells increased M2 marker genes CD206, Agr1 and Ym-1 in BMDMs, while EZH2 inhibitor treated-4T1 CM increased M2 marker genes expression more potently. In contrast, BMDMs exposed to the CM from EZH2 knockdown 4T1, showed decreased CD206, Agr1 and Ym-1 (Fig. 2E). Meanwhile, compared with co-culture with CM of parent 4T1 cells, EZH2-deleted 4T1 CM caused a nine-fold decrease of M2 macrophages migration in transwell assay, whereas the mobility of M2 macrophages was significantly enhanced when co-cultured in the CM from EZH2 inhibitor-treated 4T1 (Fig. 2F). The protein levels of these macrophage markers were also confirmed by western blot (Fig. 2G). Consistent results were observed in the MDA-MB-231/THP-1-derived M2 macrophages co-culture system. M2 markers in THP-1 were up-regulated or down-regulated when cultured with CM from MDA-MB-231 treated with EZH2 inhibitor or knockdown cells, respectively (Fig. S2A). Also, CM from EZH2 inhibitor treated MDA-MB-231 increased M2 macrophage migration; while EZH2 knockdown-MDA-MB-231 CM inhibited the migration (Fig. S2B). We next evaluated the migratory behavior of naïve BMDM and M0 THP-1-derived macrophages in response to the CM derived from cells treated with EZH2 inhibitor and siRNA, which showed a consistent change with that of M2 macrophages (Fig. S2C). And the flow cytometry results showed that the percentage of M2 macrophages (CD206^+^F4/80^+^) was increased when naïve BMDM were co-cultured with EPZ-6438-treated 4T1 cells; whereas decreased when co-cultured in 4T1 siEZH2 CM compared with siNC CM (Fig. S2D). Since tumor cells and macrophages share a common TME, it’s very likely that the drugs we use to treat tumor cells also target macrophages in the microenvironment. Thus we tested the direct effect of EZH2 inhibitors on macrophage polarization. As shown in Fig. S2E, F, EPZ-6438 and GSK126 alone could facilitate M0 macrophage polarizing toward M2 macrophages, though the induction was not very strong, which indicated that the driving force of M2 macrophage induction came from the interaction with tumor cells. Overall, these observations suggested that the crosstalk between TAMs and EZH2 inhibitor-treated BC cells induces differentiation of macrophages towards a M2-type state.

### EZH2 regulates the polarization of TAMs by CCL2 releasing

**Fig. 3:**
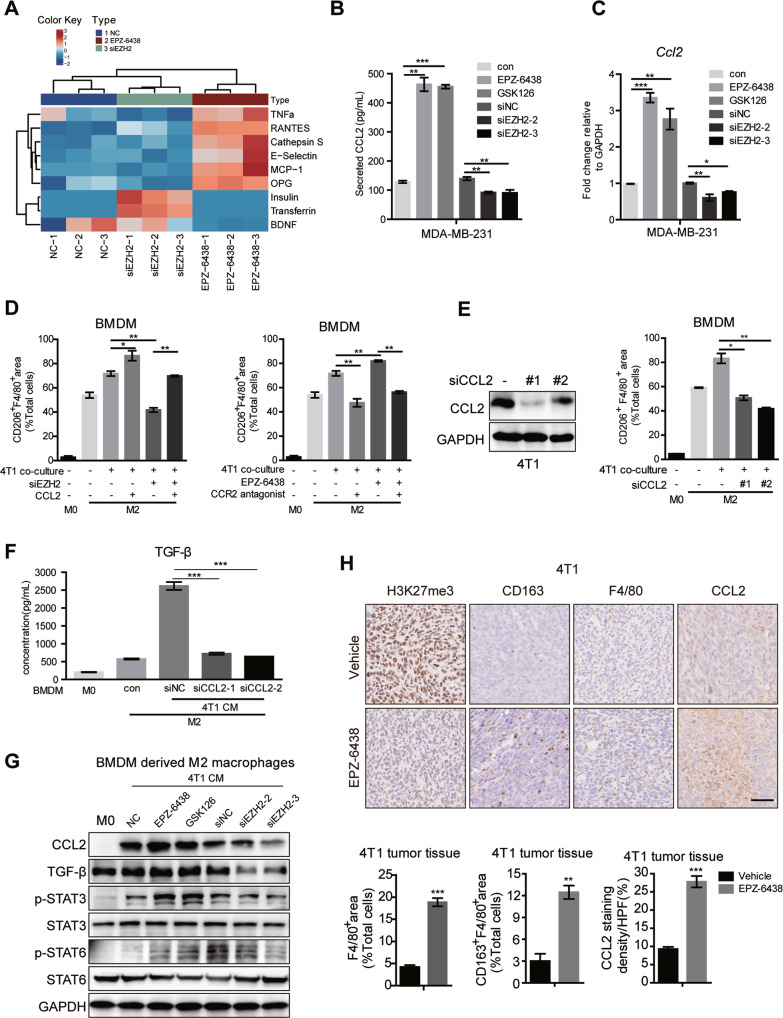
CCL2 secreted by BC cells manipulates macrophage polarization. **A** Cytokine array of cell supernatants from MDA-MB-231 treated with NC, siEZH2 or EPZ-6438 for 48 h (Human Cytokine Array GS440, RayBiotech, part of the result). **B** The concentration of CCL2 in the supernatant of MDA-MB-231 treated with siNC, siEZH2 or EPZ-6438, GSK126 for 48 h were detected by ELISA analysis. **C** The mRNA level of *Ccl2* in MDA-MB-231 treated with siNC, siEZH2 or EPZ-6438, GSK126 for 48 h were detected by RT-qPCR. **D** BMDM were co-cultured with CM from 4T1 treated with siEZH2 and CCL2, or EPZ-6438 and CCR2 antagonist RS 504393 for 48 h. Percentages of CD206^+^F4/80^+^ macrophages were analyzed by flow cytometry. BMDM were co-cultured with CM from 4T1 treated with siNC or siCCL2 for 48 h. Percentages of CD206^+^ F4/80^+^ macrophages were analyzed by flow cytometry **E**, the TGF-β level in the supernatant of BMDM were analyzed by ELISA **F** and protein levels of CCL2, TGF-β, p-STAT3 and p-STAT6 were analyzed by western blot **G**. **H** Immunohistochemistry staining of H3K27me3, CD163, F4/80 and CCL2 in the tumor tissue from EPZ-6438 or vehicle treated 4T1 xenograft. Scale bar, 50 μm. Percentages of F4/80^+^ or CD163^+^F4/80^+^ macrophages and the positive staining of CCL2 in IHC was quantified and the shown were representative of replicates. Statistical significance was addressed using unpaired, two‐tailed Student’s t‐test, *p < 0.05, **p < 0.01, ***p < 0.001, NS not significant. The statistical significance was calculated compared to the negative control (NC) in each group. The graphs were shown as means ± SD, *n* = 3 independent experiments.

To unravel the driving regulation mechanism of the opposite effects of EZH2 depletion and inhibition on TAMs polarization, a human cytokine array (GSH-CAA-440, RayBiotech, 440 human cytokines) was used to screen the secretome from EPZ-6438 or EZH2 siRNA treated MDA-MB-231 cells. CCL2, CCL5, E-Selectin, Cathepsin S, TNFα and osteoprotegerin (OPG) were significantly increased in CM from EPZ-6438-treated cells compared with those in siEZH2-treated CM (Fig. 3A). And CCL2 was ranked the first among them, which has been reported to be significantly correlated with macrophage infiltration and poor prognosis in human breast cancer [39]. We confirmed the array result by ELISA (Fig. 3B), western-blot (Fig. S3A), and RT-qPCR (Fig. 3C) in EZH2 inhibited or depleted MDA-MB-231 cells. Loss of EZH2 dramatically reduced CCL2 expression in the tumor cells, while EZH2 inhibitor conducted an opposite effect. In BMDM and 4T1 co-cultured system, we got the similar results. ELISA showed that CCL2 was increased in the medium of BMDM co-cultured with EZH2 inhibitors treated-4T1, and decreased in EZH2-deleted 4T1 co-cultured with BMDM (Fig. S3B). We also assessed the direct effect of EPZ-6438 on CCL2 secretion by M0 and M2 macrophages, which showed that EZH2 inhibitor alone could elevate the level of CCL2 in macrophages, but not nearly as high as that in the co-culture system (Fig. S3C). So the CCL2 in TME was produced by both tumor cells and TAMs, with the former one playing a major role.

We further explored the impact of CCL2 on TAMs polarization and migration. Firstly, deletion of EZH2 using siRNAs showed that the induction of M2 polarization was inhibited, which could be rescued by CCL2 supplementation (Fig. 3D). And Bindarit, a CCL2 synthesis inhibitor diminished the expression of CCL2 upregulated by EZH2 inhibitors and repressed M2 macrophage polarization in the co-culture system (Fig. S3D, F). The CCR2 antagonist RS 504393 could also counteract the effect of EPZ-6438 in promoting M2 polarization (Fig. 3D). Furthermore, genetic ablation of CCL2 using siRNAs to mimic the treatment with siEZH2 or CCR2 antagonist, also showed that the number of CD206^+^F4/80^+^ cells was suppressed (Fig. 3E, Fig. S3G, H), and TGF-β, a hallmark of M2-type cytokines was reduced (Fig. 3F). Given that transcription factors such as STAT6, STAT3 and peroxisome proliferator-activated receptor γ (PPARγ) play key roles in IL-4 and IL-13 induced M2 polarization [3], we assessed whether these pathways were involved in EZH2-regulated macrophage differentiation. The phosphorylation of STAT6 and STAT3 and the induction of PPARγ expression triggered by IL-4 and IL-13 were enhanced by EZH2 inhibitors but significantly restrained by EZH2 depletion or CCL2 knockdown in the co-culture system (Fig. 3G and Fig. S3I). Moreover, we examined the phenotype of the macrophages and the expression of CCL2 in EZH2 inhibitor-treated 4T1 tumor tissues by IHC. As shown in Fig. 3H, compared with the vehicle tumors, the population of CD163^+^F4/80^+^cells and CCL2 level were obviously upregulated in EPZ-6438-treated 4T1 tumors, where the density of the trimethylation of H3K27 (H3K27me3) was greatly reduced.

To further confirm the protumoral role of CCL2, we depleted CCL2 in MDA-MB-231 and 4T1 cells by lentivirus infection and performed in vivo tumor growth and lung metastasis assay. Compared with shCon-MDA-MB-231, loss of CCL2 significantly delayed tumor growth in vivo. And there was almost no difference in tumor growth between EPZ-6438 treated group and the untreated group in CCL2 silencing MDA-MB-231 in vivo model (Fig. S4A). CCL2 knockdown in 4T1 cells prominently reduced lung colonization and attenuated the inducing effect of EPZ-6438 in the metastasis model (Fig. S4B). Confocal images of the tumor tissues revealed the obvious downregulation of the CD206^+^F4/80^+^ macrophages accumulation in shCCL2 tumors. And depletion of CCL2 totally reversed EPZ-6438’s induction effect on M2 polarization (Fig. S4C, D). These data indicated that CCL2 is vital for the crosstalk between BC cells and TAMs, and mediates TAMs M2 polarization and infiltration via EZH2 inhibitors. Blocking this pathway may improve the anti-tumor effect of EZH2 inhibitors.

### miR-124-3p is a post-transcriptional regulator of CCL2 expression

**Fig. 4:**
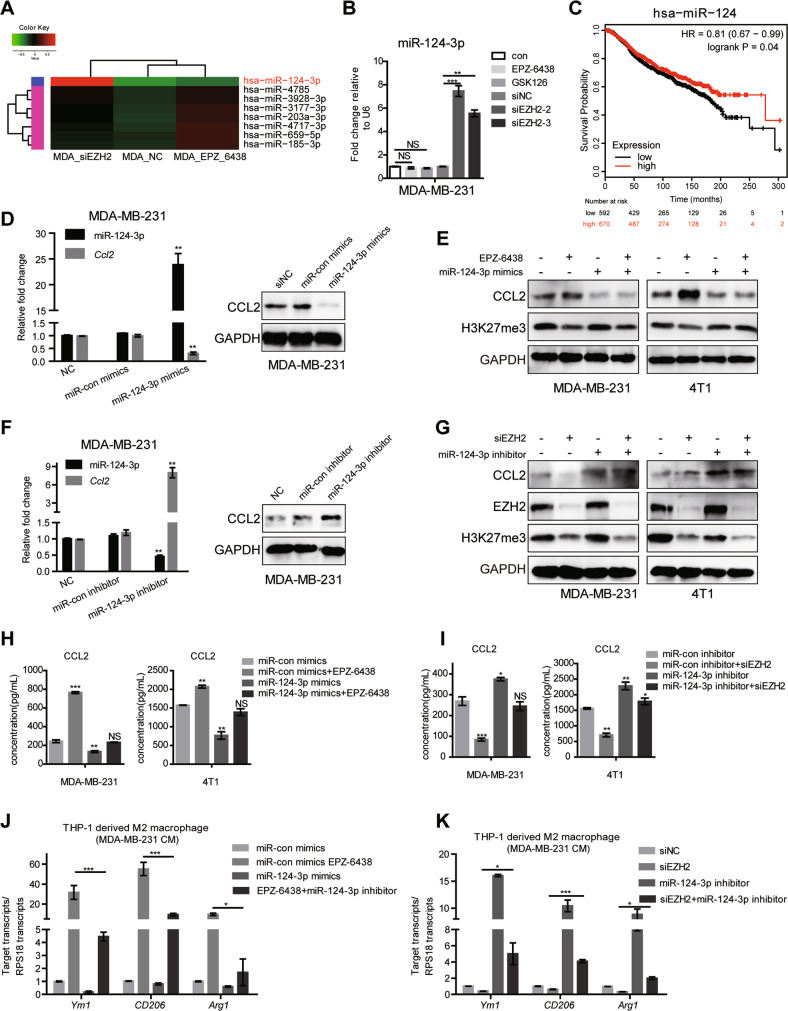
EZH2 inhibition induces miR-124-3p level and regulates CCL2 expression. **A** Differentially expressed miRNAs in MDA-MB-231 EZH2 siRNA or inhibitor-treated cells screened by miRNA-seq (part of the result), aligned according to their expression levels, red showing upregulation in MDA-MB-231 siEZH2 cells. **B** The miR-124-3p level was analyzed by RT-qPCR in MDA-MB-231 cells treated with EZH2 inhibitors or siRNAs for 48 h. U6 was included as a control. *n* = 3. **C** Relationship between has-miR-124 expression level and overall survival of patients with breast cancer (http://kmplot.com). **D** The miR-124-3p and CCL2 level were analyzed by RT-qPCR and western blot in MDA-MB-231 cells treated with miR-124-3p mimics or con-mimics for 48 h. U6 was included as a control. **E** Western blot showed miR-124-3p mimics repressed CCL2 level induced by EZH2 inhibitors in MDA-MB-231 and 4T1 cells. **F** The miR-124-3p and *Ccl2* level were analyzed by RT-qPCR and western blot in MDA-MB-231 cells treated with miR-124-3p inhibitor or con-inhibitor for 48 h. U6 was included as a control. **G** Western blot showed miR-124-3p inhibitor induced CCL2 level repressed by EZH2 knockdown in MDA-MB-231 and 4T1 cells. The CCL2 concentration was tested by ELISA in MDA-MB-231 and 4T1 cells treated with EPZ-6438 and miR-124-3p mimics **H**, or siEZH2 and miR-124-3p inhibitor **I**. RT-qPCR tested the expression of M2-type markers in THP-1-derived M2 macrophages after being co-cultured with MDA-MB-231 cells treated with EPZ-6438 and miR-124-3p mimics **J**, or siEZH2 and miR-124-3p inhibitor **K**. The shown were representative of replicates and the experiments were repeated three times. Statistical significance was addressed using unpaired, two‐tailed Student’s t‐test, *p < 0.05, **p < 0.01, ***p < 0.001, NS not significant. The statistical significance was calculated compared to the negative control (NC) in each group.

Next, we sought to understand the molecular mechanisms by which EZH2 regulates CCL2 expression. Chromatin immunoprecipitation (ChIP) assay verified that EZH2 inhibitors resulted in a marked reduction of H3K27me3 recruitment in *Ccl2* promoter region (Fig. S5A), giving clear evidence that inhibition of EZH2 histone methyltransferase activity accounts for *Ccl2* transcription induction in BC cells. However, the opposite effect of EZH2 interference on CCL2 expression strongly suggested that there are other regulatory mechanisms. MicroRNAs (miRNAs) are endogenous small noncoding RNAs with approximately 20–24 nt RNAs, which modify gene expression by pairing to the 3′ untranslated region (UTR) of specific protein-coding genes to direct their posttranscriptional repression [40]. Previous studies have reported that miRNA can control various cytokines production in the immune system [41]. Therefore, CCL2 may also be targeted by EZH2 regulated miRNAs. So, we constructed three small RNA libraries (EPZ-6438, siEZH2 and non-treated group), which were sequenced by Illumina HiSeq 2000 to identify miRNA transcriptome differences. As partial results shown in Fig. 4A, the level of miR-124-3p remarkably increased in the siEZH2-treated group compared with the other two groups and the result was confirmed by RT-qPCR (Fig. 4B). It has been revealed that miR-124-3p functions as a tumor suppressor in BC by targeting CBL and MGAT5 [42, 43]. We examined the prognostic value and clinical significance of miR-124-3p expression in BC using the Kaplan–Meier (KM) plotter miRNA breast cancer online database (http://kmplot.com/analysis/). Among datasets included in the KM plotter, we employed the Molecular Taxonomy of Breast Cancer International Consortium (METABRIC) cohort dataset, which analyzes data from 1262 patients, to assess the association between miR-124-3p and overall survival (OS) in BC patients. Consistent with previous findings, OS of patients with low level miR-124 was significantly lower than that of patients with high level miR-124 in all 1262 patients (HR = 0.81, 95% CI = 0.67–0.99, *p* = 0.04) (Fig. 4C).

What’s coincidental is that, CCL2 may be one target of miR-124-3p according to some studies [44, 45] and the miR-124-3p-binding site in 3’UTR region of *Ccl2* was predicted by informatics tools (TargetScan 7.2 and PicTar) (Fig. S5B). The treatment with miRNA mimics of miR-124-3p strikingly reduced the expression of CCL2 at mRNA and protein levels in MDA-MB-231 cells (Fig. 4D, E), while miR-124-3p inhibitor dramatically increased the level of CCL2 (Fig. 4F, G). In addition, the miR-124-3p mimics inhibited the stimulation of CCL2 by EPZ-6438, while the inhibitor of miR-124-3p partially reversed the CCL2 decrease induced by EZH2 deletion (Fig. 4E, G–I). The findings demonstrated the suppressive role of miR-124-3p in CCL2 expression. Mimics of miR-124-3p repressed the CCL2 secretion, resulting in decreased THP-1 derived-M2 macrophages polarization and impaired mobility of macrophages in the co-culture system with MDA-MB-231 (Fig. 4J and Fig. S5C). On the contrary, miR-124-3p inhibitor elevated CCL2 and reversed the repressive role of EZH2 knockdown in M2 macrophage polarization and migration (Fig. 4K and Fig. S5C). The similar results were observed in the 4T1/BMDM co-culture system (Fig. S5D). Moreover, western blot showed that the change of CCL2 together with M2 macrophage hallmarks, CD206, Arg-1 and p-STAT6 were consistent with macrophages polarization (Fig. S5E).

### miR-124-3p is suppressed by DNA hypermethylation mediated by EZH2 and DNMT1

**Fig. 5:**
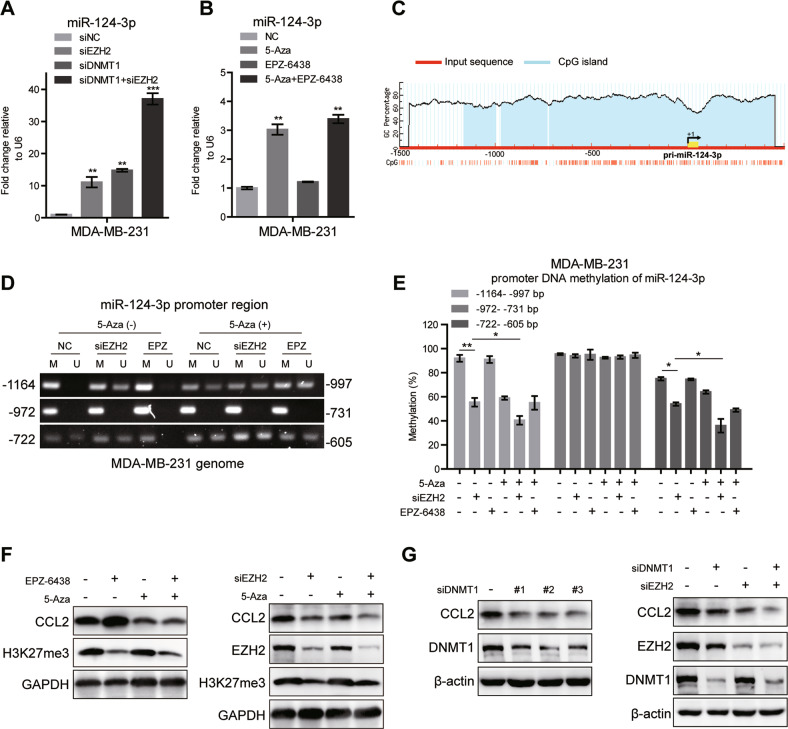
miR-124-3p is regulated by EZH2 and DNMT1 mediated DNA methylation. **A** The miR-124-3p level was analyzed by RT-qPCR in MDA-MB-231 cells treated with EZH2 siRNA or/and DNMT1 siRNA for 48 h. U6 was included as a control. *n* = 3. **B** The miR-124-3p level was analyzed in MDA-MB-231 cells treated with 5 μM 5-Aza-CdR (5-Aza) or/and EPZ-6438 for 48 h. U6 was included as a control. *n* = 3. **C** Modified output of MethPrimer program (Li and Dahiya, 2002). Coordinates are given in relation to the transcription start site (TSS). A large CpG island (−1200 to +450 bp, blue region) is evident in the upstream of the gene. Vertical lines indicate relative positions of CpG dinucleotides. **D** 5-Aza-CdR (5-Aza) and EZH2 siRNA treatment decreased the DNA methylation level of miR-124-3p promoter region (−1164 to −997 and −722 to −605bp) in MDA-MB-231 cells analyzed by MSP. The relative percentages of the methylation were quantified due to the PCR results **E**. **F** The CCL2 levels were analyzed by western blot in MDA-MB-231 cells treated with 5-Aza and EZH2 siRNA or 5-Aza and EPZ-6438 for 48 h. **G** The CCL2 levels were analyzed by western blot in MDA-MB-231 cells treated with siDNMT1 and siEZH2 for 48 h. The shown were representative of replicates and the experiments were repeated three times. In (**A**, **B** and **E**), all data were shown as mean values ± SD (*n* = 3). Statistical significance was addressed using unpaired, two‐tailed Student’s *t*‐test, *p < 0.05, **p < 0.01, ***p < 0.001, NS not significant. The statistical significance was calculated compared to the negative control (NC) in each group.

The opposite effects of EZH2 knockdown and inhibition on miR-124-3p suggested that EZH2-mediated regulation of miR-124-3p expression should be independent of its histone methyltransferase activity. It has been reported a strong correlation between promoter methylation and the expression level of miR-124-3p [46]. Increasing evidence points out that DNA methyltransferase 1 (DNMT1), the key enzyme that ensures DNA methylation can be recruited by EZH2 to catalyze hypermethylation of many miRNA promoters [47, 48]. We abolished DNMT1 and EZH2 expression by siRNA and found that knockdown either of the two genes alone induced the expression of miR-124-3p mRNA, and deletion of these two genes together had a more obvious induction in MDA-MB-231 (Fig. 5A), indicating that DNA methylation contributed to the downregulation of miR-124-3p in BC. What was more, EPZ-6438 hardly affected miR-124-3p mRNA, while 5-Aza-2′-deoxycytidine (5-Aza-CdR, or 5-Aza), a DNA methylation inhibitor which is frequently used to induce demethylation, significantly upregulated miR-124-3p mRNA in MDA-MB-231 cells, which was almost equivalent to the effect of the combination of 5-Aza and EPZ-6438 (Fig. 5B). To verify the effect of DNA methylation on miR-124-3p expression, the bioinformatics software MethPrimer was used to predict the range of CpG islands in the core promoter region of miR-124-3p. It showed that there was a large area of an independent CpG islands in the promoter region, which may serve a crucial role in the expression of the miR-124-3p (Fig. 5C). Genomic bisulfite sequencing was performed and revealed that the methylation level on two CpG islands, one harboring 168 CpG dinucleotides (−1164 to −997) and the other harboring 118 CpG dinucleotides (−722 to −605), was decreased after cells treated with 5-Aza or siEZH2 alone or both (Fig. 5D, E). Furthermore, co-immunoprecipitation (co-IP) assay showed that EZH2 physically interacted with DNMT1 in MDA-MB-231 cells, whereas the interaction was markedly reduced following EZH2 knockdown (Fig. S5F), suggesting that EZH2 may be required for DNMT1 binding to miR-124-3p. Further, as expected, CCL2 expression was prominently reduced by treated with 5-Aza, siDNMT1 or siEZH2 alone, accompanied with the upregulation of miR-124-3p transcription (Fig. 5F, G). And the decrease of CCL2 was more dramatic in the combination treatments, 5-Aza plus siEZH2 or siDNMT-1 plus siEZH2 (Fig. 5F, G). Meanwhile, EPZ-6438 increased CCL2 expression, which could be abolished by 5-Aza treatment (Fig. 5F). All the findings strongly suggested that the level of miR-124-3p is regulated by EZH2 and DNMT1 through DNA methylation, thus manipulating the expression of its target protein CCL2. This process requires EZH2, but is independent of its histone methyltransferase activity.

### IL-10 secreted by TAMs mediates the crosstalk between TAMs and BC cells

**Fig. 6:**
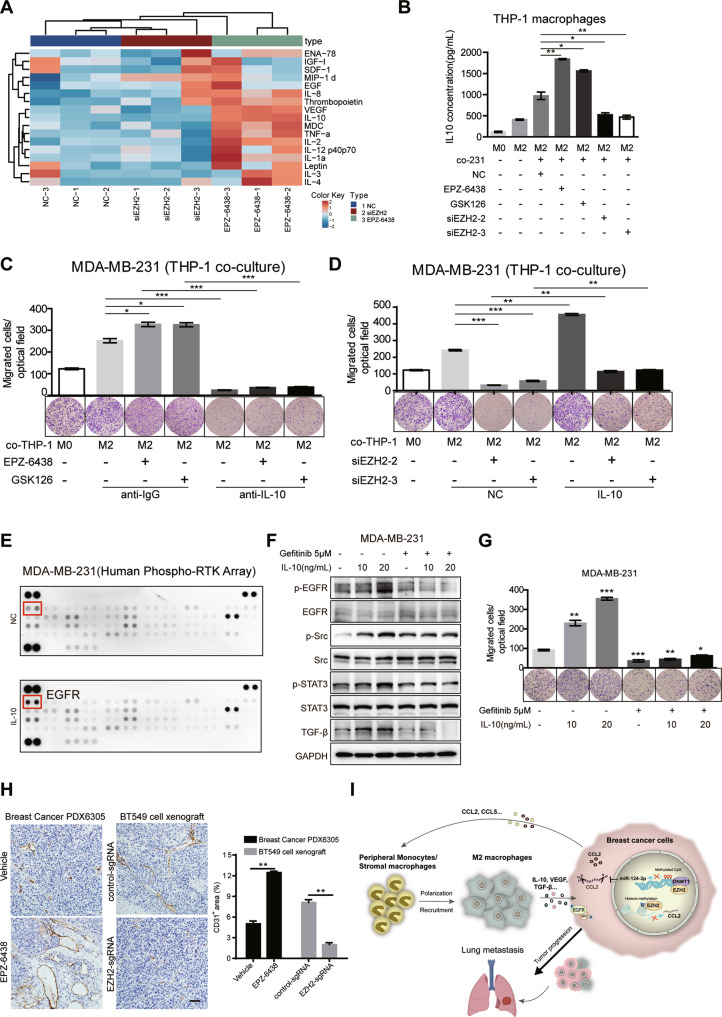
IL-10 secreted from M2 TAMs promotes BC cell metastasis in the co-culture system. **A** Cytokine array of cell supernatant from THP-1 derived M2 macrophages co-cultured in different MDA-MB-231 CM as indicated (AAH-CYT-G3, RayBiotech, 42 human cytokines, part of the results was shown). **B** The IL-10 concentrations of cell supernatant from THP-1 derived M2 macrophages co-cultured in different MDA-MB-231 CM as indicated were analyzed by ELISA. **C** Transwell assay for MDA-MB-231 cells treated with EZH2 inhibitors were plated on the upper cell culture inserts, with culture medium alone (NC), or IL-4-activated THP-1 cells (M2 macrophages) plated in the lower chambers, in the presence or absence of an anti-IL10 antibody at 10 ng/ml, or an isotype-matched IgG control (IgG). **D** Transwell assay for MDA-MB-231 cells treated with EZH2 siRNAs were plated on the upper cell culture inserts, with culture medium alone (NC), or IL-4-activated THP-1 cells (M2 macrophages) plated in the lower chambers, in the presence or absence of IL-10 at 20 ng/ml. **E** MDA-MB-231 cells were treated with IL-10 at 20 ng/ml for 24 h, and subjected to human phospho-RTK array (R&D, ARY001B). Each kinase is spotted in duplicate. The pairs of dots in each corner (with the exception of the negative control pair at the lower left corner) are positive controls. The upregulated responding kinases were shown in red frame, with the name of the corresponding kinases. MDA-MB-231 cells were treated with or without 5 μM Gefitinib for 48 h before IL-10 stimulation as indicated for 24 h. The migration capacity was analyzed by transwell assay **F** and the phosphorylation of EGFR, Src and STAT3 and TGF-β level were tested by western blot **G**. **H** IHC staining of CD31 in the tumor tissue from EPZ-6438 or vehicle treated PDX6305 model and BT549 control or EZH2 sgRNA xenografts. Scale bar, 50 μm. The positive area of CD31 in IHC was quantified and the shown were representative of replicates. Statistical significance was addressed using unpaired, two‐tailed Student’s t‐test, *p < 0.05, **p < 0.01, ***p < 0.001, NS not significant. **I** Model describing that EZH2 in BC promotes tumor progression and metastasis by increasing CCL2-dependent recruitment and polarization of M2 TAMs, which reciprocally secrete IL-10, VEGF and other cytokines to activate EGFR signaling in cancer cells (the lung is created from BioRender.com).

Immune mediators secreted by TAMs could possibly be the component of intriguing related signaling pathways in cancer cells to influence cancer progression. We therefore analyzed production of multiple cytokines/chemokines utilizing a commercially available human antibody-based array (AAH-CYT-G3, RayBiotech, US) which revealed a robust increase in the level of IL-10, IL-2, TNFα, IL-1α and vascular endothelial growth factor (VEGF) levels in THP-1 derived macrophages cultured in EPZ-6438-treated cells CM compared with siEZH2-treated cells CM (Fig. 6A). IL-10 was the most dramatically upregulated cytokine, which has been proved to be related to the pathogenesis and progression of human breast cancer [49]. The concentration of IL-10 in the supernatant of THP-1 cultured in the CM was detected by ELISA, which was consistent with the array result (Fig. 6B). Inducible ablation of IL-10 was achieved in cultured THP-1-derived M2 macrophages treated with IL-10 neutralizing antibody. And then, compared with the control group, these macrophages could not promote MDA-MB-231 cells migration (Fig. 6C), a process important for metastasis. On the contrary, IL-10 supplementation rescued the migration reduction caused by EZH2 depletion (Fig. 6D). These data indicated that M2 macrophage induced by EZH2 inhibitors secrete IL-10, which acts as a potent chemoattractant for BC cells in return.

To determine the mechanism of IL-10 secreted by TAMs to promote tumor cells mobility, we analyzed the phosphorylated proteins of tumor cells cultured in conditioned medium with or without IL-10 by human phospho-RTK array (R&D system). Among the differentially affected proteins, phosphorylation of epidermal growth factor receptor (EGFR) was highly increased in tumor cells treated with IL-10 (Fig. 6E). The array result was confirmed by western blot analysis, p-EGFR, together with p-Src, p-STAT3 and TGF-β were upregulated upon IL-10 treatment in MDA-MB-231 cells (Fig. 6F), which may contribute to the corresponding cell motility all together. Gefitinib, an EGFR inhibitor diminished the activation of these protein induced by IL-10, and inhibited tumor cells migration (Fig. 6F, G). Thus, these data indicated that IL-10 synthesized by TAMs treated with EZH2 inhibitor CM is critical for enhancing the migration of tumor cells in return. Mechanistically this is at least in part through activating EGFR and Src signaling.

We also noted in particular that VEGF level increased a lot in the supernatant of THP-1 derived macrophages cultured in EPZ-6438-treated cells CM, but decreased in that cultured in siEZH2-treated CM. It is well established that the pro-metastatic function of TAMs is tightly associated with their ability to drive the “angiogenic switch” in primary breast cancer and precede the transition to malignancy [50]. The development of a high-density vascular network is induced by multiple angiogenic growth factors such as VEGF and proteinases secreted by these infiltrated macrophages [51]. Thus, we speculated that EZH2 inhibitor treatment might promote tumor metastasis in association with augmented tumor angiogenesis through TAMs-secreted VEGF. Specified cell adhesion molecules such as PECAM-1 (CD31) involved in the subsequent events of endothelial cell differentiation and angiogenesis are recognized as markers of vasculogenesis [52]. We evaluated the vascularity by measuring the immunoreactivity score of CD31-positive cells. IHC staining using anti-CD31 demonstrated that tumors from EPZ-6438-treated groups showed a higher vascular density than those from vehicle controls (Fig. 6H). While the percentage of CD31^+^ area in EZH2 knockdown tumor tissues was much lower than that in control tumors due to a reduced secretion of VEGF by TAMs (Fig. 6H). Collectively, we suggested that tumor angiogenesis should be evaluated as an additional mechanism underlying the pro-metastatic activity of TAMs in mice treated with EZH2 inhibitors.

## Discussion

TAMs are pivotal immune cells in the TME that orchestrate the crosstalk between inflammation and cancer to facilitate tumor progression and metastasis [53]. The role of EZH2 or EZH2 inhibitors in breast cancer development by affecting macrophage polarization has not been uniformly determined. Some research show that EZH2 inhibition leads to significant reduction of M2 markers (CD206) and elevation of M1 markers (TNFα and iNOS) in TAMs co-cultured with murine glioblastoma cells [54, 55]. But another paper pointed out that the EZH2 inhibitor GSK343 induced the transformation of M1 macrophages into M2 macrophages [56]. In addition, EZH2 has been shown to enhance the expression of CCL5 to promote recruitment of macrophages and invasion in lung cancer, although the mechanism was not clearly identified [57]. Suppressing EZH2 activity by EZH2 inhibitor GSK126 resulted in more myeloid-derived suppressor cells (MDSC) and less CD4^+^ and IFNγ^+^CD8^+^ T cells accumulated in tumors, which are key components of anti-tumor immunity, suggesting that EZH2 inhibitors may exhibit antitumor efficacy by modulating tumor immune microenvironment [58]. Recent study reports that EZH2 inhibitor EPZ-6438 triggers malignant pleural mesothelioma cells to express high level of different monocytes chemoattractants, including CCL2 and CSF1, which lead to increased monocytes recruitment in tumor spheroids as well as M2 TAMs differentiation [59]. However, the role of EZH2 in breast cancer development by affecting macrophage polarization has not been uniformly determined. In this study, in the context of BC, we proved that EZH2 depletion and EZH2 inhibitors had completely opposite effects on TAMs polarization. We confirmed that EZH2 converts TAMs to a tumor-promoting M2 phenotype to facilitate BC progression and metastasis. To begin with investigation into clinical BC patient samples, we identified a significant positive correlation between EZH2 expression and M2 macrophage accumulation. We also found that EZH2 knockdown eliminated TAMs infiltration in vitro and in vivo. However, the effect of EZH2 inhibitor-treated cells on TAMs is almost completely opposite. EZH2 inhibitors and CM from EZH2 inhibitor-treated cells activated TAMs towards M2 polarization in vitro and in vivo, which might help us understand why EZH2 inhibitors do not achieve benefits in preclinical breast cancer models.

Another important finding in our study is that EZH2 can epigenetically regulate CCL2 expression through transcriptional and post-transcriptional ways, resulting in the opposite impact on TAMs polarization. EZH2 inhibitors reduced H3K27me3 level at the promoter region of CCL2, increased CCL2 transcription and secretion in BC cells, which activated TAMs to M2-type transition. Inhibition of CCL2 could break this loop and reverse the enhanced BC cell tumor proliferation and migration caused by EZH2 inhibitors, which suggested that combinational inhibition of CCL2 with EZH2 inhibitors may be beneficial to the treatment of breast cancer. Although loss of EZH2 also decreased H3K27me3 on CCL2 promoter, we found that there was another pivotal regulatory mechanism independent of the methyltransferase activity of EZH2. It has been reported that the DNA methylation mediated by DNMT1 and EZH2 silences a lot of genes including miR-200b/a/429 expression and promotes tumor progression [60], which may help explain why EZH2 depletion has different effects from EZH2 inhibitors sometimes. EZH2 serves as a recruitment platform for DNA methyltransferases (DNMTs), and influences DNA methylation directly by interaction with DNMTs [61]. In our case, the CCL2 regulation process is also mediated by EZH2 and DNMT1. By cooperating with DNMT1, EZH2 knockdown led to DNA demethylation and consequently upregulated miR-124-3p, which suppressed its target CCL2 expression in breast cancer cells, leading to TAMs M2 polarization restraint. These findings give the mechanistic and translational insight into the EZH2-mediated modulation of tumor microenvironment that promotes breast cancer progression, and emphasize the importance of understanding the function of EZH2 beyond its enzymatic role. In addition, the regulation of tumor-macrophage interactions by EZH2-miR-124-3p-CCL2 axis as uncovered in our study demonstrated that blockade of cytokines not only had direct intrinsic inhibitory effect on tumor cells, but also educated the TME toward an anti-tumor phenotype by altering the relative proportion between pro-tumor and anti-tumor macrophages or other immune cells. These data also indicated that the combinatorial anti-CCL2 therapy might improve the efficacy of EZH2 inhibitors in breast cancer treatment, although further evidence is needed.

Multiple studies have demonstrated the critical role of the tumor microenvironment (TME) in tumor progression, which serves as the basis of various kinds of immunotherapy. Among its complex components, cancer-associated fibroblasts (CAFs) are major providers of extracellular matrix molecules and key contributors to inflammation, implicated in tumor growth, metastasis, angiogenesis, immune cell reprogramming and therapeutic resistance [62, 63]. It has been reported that CAFs promote hepatocellular carcinoma angiogenesis and metastasis through VEGF-induced upregulation of EZH2 and subsequent downregulation of vasohibin 1, a negative regulator of angiogenesis, emphasizing an important role of EZH2 in mediating the function of CAFs [64]. It has also been revealed that in hepatic tumor cells, genetic or pharmacological inhibition of EZH2 induces the transcription and secretion of chemokine CXCL10, which is necessary and sufficient for stimulating NK cell migration [65]. Consistently, other study reports that EZH2-mediated H3K27me3 and DNMT1-mediated DNA methylation inhibit the expression of tumor-derived T helper 1 (TH1)-type chemokines CXCL9 and CXCL10, and treatment with epigenetic modulators gets rid of the suppression, promotes tumor-infiltrating effector T-cells and strengthens the therapeutic efficacy of programmed death-ligand 1 (PD-L1) checkpoint blockade [66]. Moreover, EZH2 and DNMT1 expression in tumor are negatively correlated with CD8^+^ T cells tumor-infiltration and patient outcomes. Collectively, EZH2 and DNMT1 have been shown to regulate the orchestration of innate and adaptive immune systems of the solid tumor microenvironment. Inactivation of EZH2 or DNMT1 exerts much immunological relevance, which may affect its anti-tumor therapeutic efficacy while enabling immune-modifying agents [67]. Interpreting the mechanism of EZH2 and DNMT1 conductor in cancer immunity throws light on the promising benefit of the combinational treatment of epigenetic-modifying drugs (such as EZH2 or DNMT1 inhibitor) and immunotherapy (such as TAMs modulator) in patients with cancer.

It has been revealed that the CCL2/CCR2 axis orchestrates monocytes undergoing phenotypic and functional transformation in response to immune cues [68]. Although shRNA-mediated downregulation of CCL2 overcomes the resistance of EZH2 inhibitors, we cannot rule out that other factors including CCL5, which participate in macrophage recruitment and polarization, are also involved in the significantly upregulated population of M2 macrophages in the EZH2-inhibited tumors based on our cytokine array results. As well, on the side of TAMs that are educated by EZH2 inhibitor-treated BC cells, the increased IL-10 secretion stimulates EGFR-SRC signaling to enhance cell motility and the increased VEGF secretion accelerates vasculature development, which contribute to tumor metastasis all together. Our findings highlight some leading molecules to identify the key role of macrophages in the tumor immunosuppression under targeted therapies and provide insight into new biomarkers and combinational strategies. There is a strong need for further research.

Taken together, this study reveals a potential explanation behind the frustrating results of EZH2 inhibitor EPZ-6438 against breast cancer and sheds light on a translatable and feasible combinational strategy to overcome it. Breast cancer cases may benefit from therapies that blockade the EZH2-CCL2/CCR2 cell revulsive axis or its amplifier IL-10-EGFR pathway for the alleviation of cancer growth and dissemination. Our results also imply that the therapeutic efficacy of antitumor drugs may be attributable to their influence on TME remodeling, and the combined application with TAMs modifiers should be warranted to achieve great clinical success.

## Availability of data and materials

The dataset supporting the conclusions of this article are included within the article and its additional file.

## Supplementary information

Supplementary Information

Supplemental Meterial

aj-checklist
