# Supplementary material for: Regulation of CCL2 by EZH2 affects tumor-associated macrophages polarization and infiltration in breast cancer
Source: Cell Death Dis. 2022 Aug 29;13(8):748. doi: 10.1038/s41419-022-05169-x (PMC9424193; doi:10.1038/s41419-022-05169-x)
Supplement: Supplementary file 1 — Supplementary Information [file 41419_2022_5169_MOESM1_ESM.docx]

**Supplementary Figures and Tables**

**
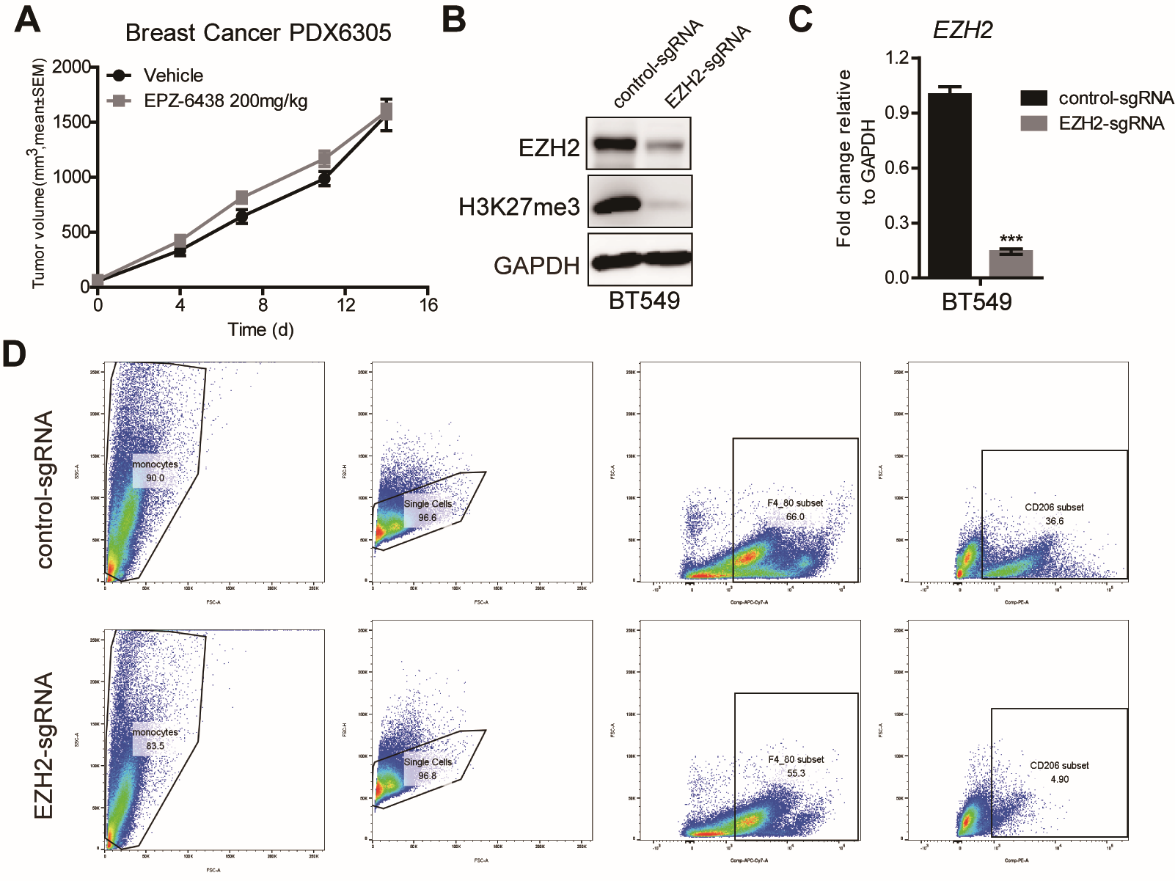
**

**Figure S1 related to Figure 1, EZH2 inhibition or depletion affects TAMs polarization**

**(A)** Tumor growth in BC PDX6305 xenograft-bearing mice following treatment with EPZ-6438 (200 mg/kg/day, oral administration, n = 6) or vehicle control (n = 6) for 14 days. Error bars represent mean ± S.E.M. The EZH2 protein levels in BT549 control-sgRNA and EZH2-sgRNA were tested by western blot **(B)** and mRNA levels were tested by RT-qPCR **(C)**. (**D)** The gating strategy of FACS performed to analyze the subsets and differentiation of M2 TAMs in the BT549 xenograft tumor tissue. In **(C)**, data were shown as mean values ± SD (n = 3). Statistical significance was addressed using unpaired, two‐tailed Student's t‐test, *p < 0.05, **p < 0.01, ***p < 0.001, NS, not significant. The shown were representative of replicates and the experiments were repeated three times.


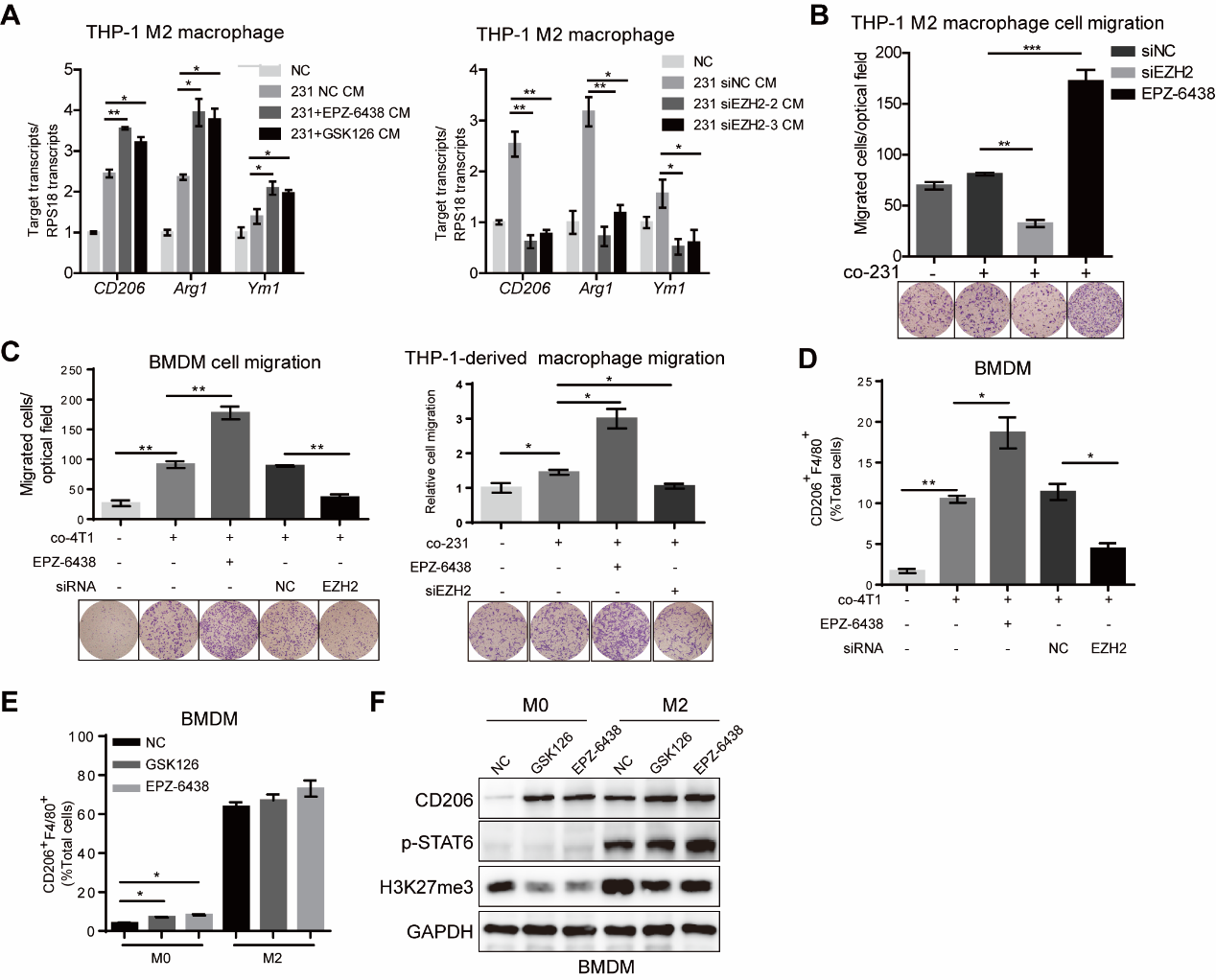


**Figure S2 related to Figure 2, EZH2 inhibitor-treated BC cells activate TAMs to a M2 phenotype**

MDA-MB-231 (231 for short) cells were transiently transfected with siEZH2 or treated with EPZ-6438. CM was used as a chemoattractant for macrophages in a transwell migration assay. (**A)** The relative transcript levels of the M2 marker genes in the CM-treated THP-1 derived M2 macrophages were determined by RT-qPCR. (**B)** The migration rates of THP-1-derived M2 macrophages with or without the co-culture of MDA-MB-231 CM were determined by transwell assay. (**C**) The migration rates of naïve BMDM and THP-1-derived M0 macrophages with or without the co-culture of 4T-1 and MDA-MB-231 CM respectively were determined by transwell assay. (**D**) Naïve BMDM were co-cultured with CM from 4T1 treated with EPZ-6438, siNC or siEZH2 for 48 hr. Percentages of CD206^+^F4/80^+^ macrophages were analyzed by flow cytometry. M0 and M2 BMDM were treated with 5 μM EPZ-6438 or GSK126 for 48 hr and the percentages of M2-type macrophages (CD206^+^F4/80^+^) were analyzed by flow cytometry (**E)**, and the M2 markers expressions were analyzed by western blot (**F**). All the graph data were shown as mean values ± SD (n = 3). Statistical significance was addressed using unpaired, two‐tailed Student's t‐test, *p < 0.05, **p < 0.01, ***p < 0.001, NS, not significant. The shown were representative of replicates and the experiments were repeated three times.


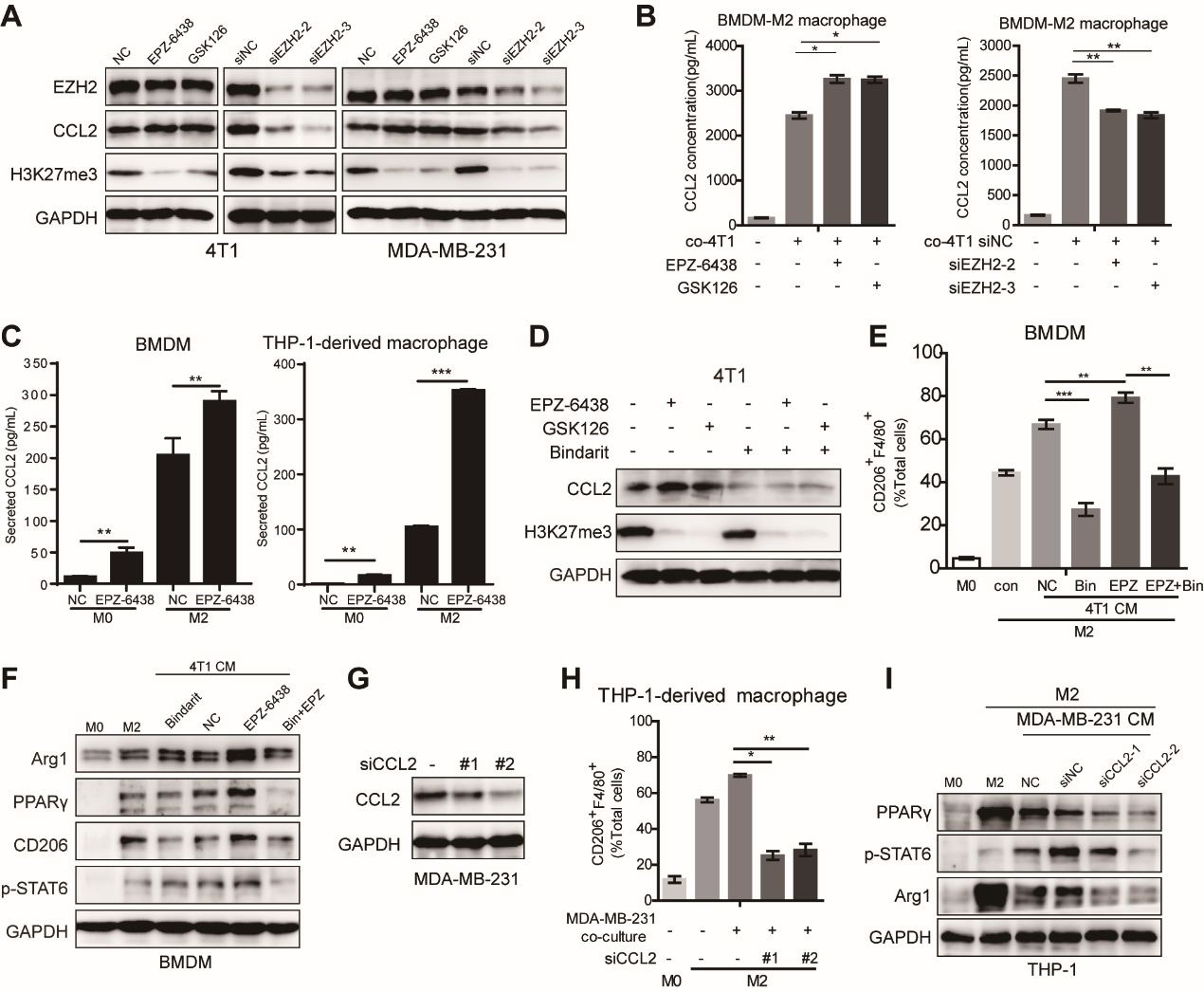


**Figure S3 related to Figure 3, CCL2 mediates the crosstalk between breast cancer cells treated with EZH2 inhibitor and macrophages in the co-culture system**

**(A)** The levels of CCL2 in 4T1 and MDA-MB-231 cells treated with EZH2 inhibitors EPZ-6438 or GSK126 and EZH2 siRNAs for 48 hr were analyzed by western blot. **(B)** The levels of CCL2 in the supernatant of BMDM-M2 macrophages in the co-culture system were detected by ELISA analysis. (**C)** The concentrations of CCL2 in the supernatant of M0 and M2 BMDM or THP-1-derived macrophages treated with or without EPZ-6438 for 48 hr were detected by ELISA analysis. **(D)** Treatment of 200 μM CCL2 inhibitor Bindarit for 24 hr suppressed the CCL2 level induced by EZH2 inhibitors in 4T1 cells. BMDM cells were co-cultured with 4T1 treated with EPZ-6438 (EPZ for short) or/and Bindarit (Bin for short) for 48 hr, and the percentages of M2-type macrophages (CD206^+^F4/80^+^) were analyzed by flow cytometry **(E)**, the M2 markers expressions were analyzed by western blot **(F)**. **(G)** Western blot showed CCL2 was knockdown by siRNAs in MDA-MB-231. After co-cultivation with CM from CCL2 knockdown MDA-MB-231, the percentages of M2-type macrophages (CD206^+^F4/80^+^) were analyzed by flow cytometry **(H)**, the M2 markers expressions were analyzed by western blot **(I)**. All the graph data were shown as mean values ± SD (n = 3). Statistical significance was addressed using unpaired, two‐tailed Student's t‐test, *p < 0.05, **p < 0.01, ***p < 0.001, NS, not significant.


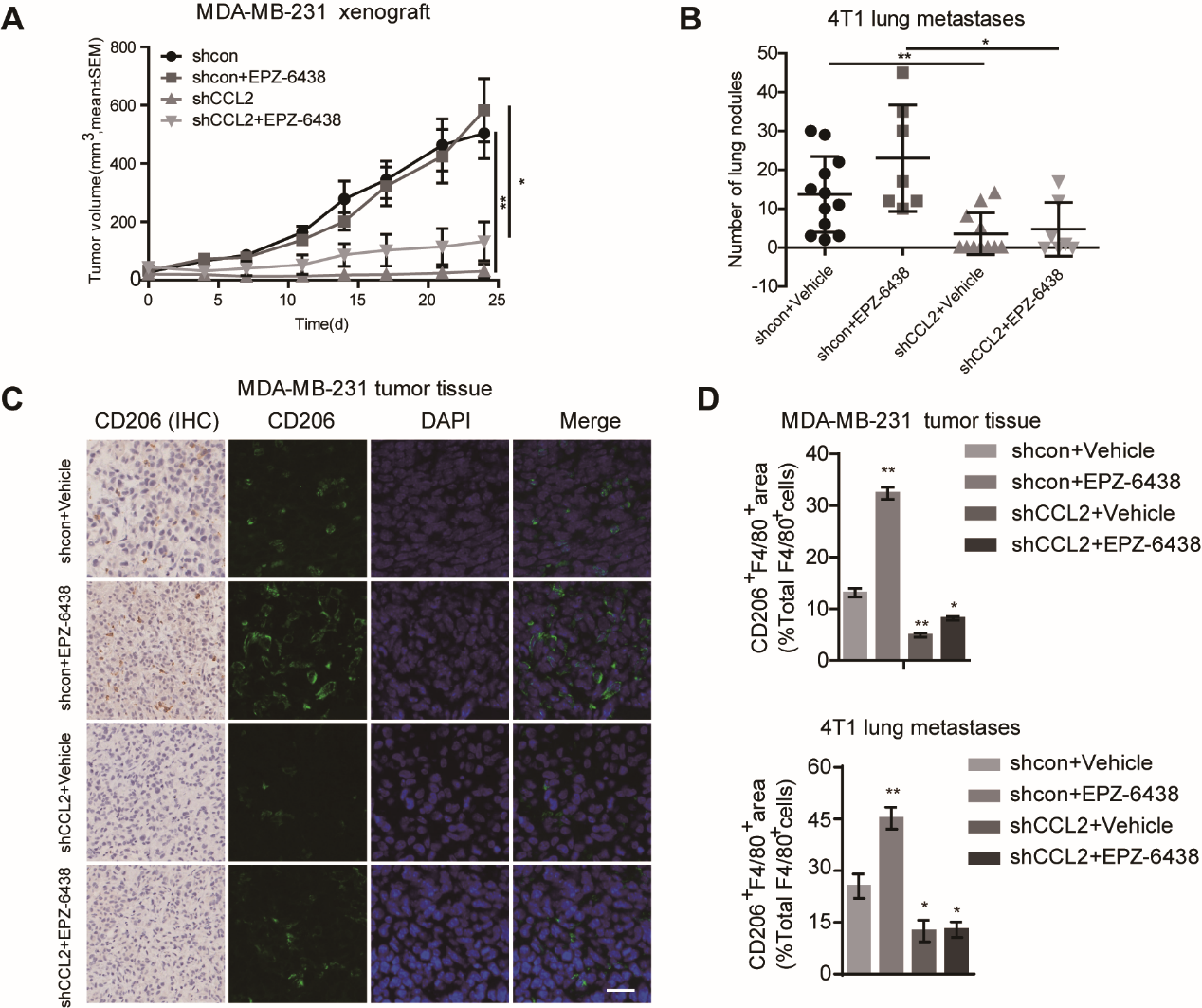


**Figure S4 related to Figure 3. CCL2/CCR2 blockade facilitates EZH2 inhibition anti-tumor effect**

**(A)** Tumor growth of MDA-MB-231 CCL2 knockdown xenograft treated with or without EPZ-6438, n = 6 in each group. **(B)** The numbers of lung metastatic foci in 4T1 CCL2 knockdown mice treated with or without EPZ-6438, n = 10 in each group. **(C)** MDA-MB-231 tumor tissues were stained for CD206 (IHC and Alexa Fluor 488). Nuclei were stained with DAPI. Representative immunofluorescence images were shown. Scale bar, 50 μm. **(D)** Percentages of CD206^+^F4/80^+^ TAMs in MDA-MB-231 tumor tissue and 4T1 lung metastases were analyzed by flow cytometry and immunofluorescence respectively. Statistical significance was addressed using unpaired, two‐tailed Student's t‐test, *p < 0.05, **p < 0.01, ***p < 0.001, NS, not significant.


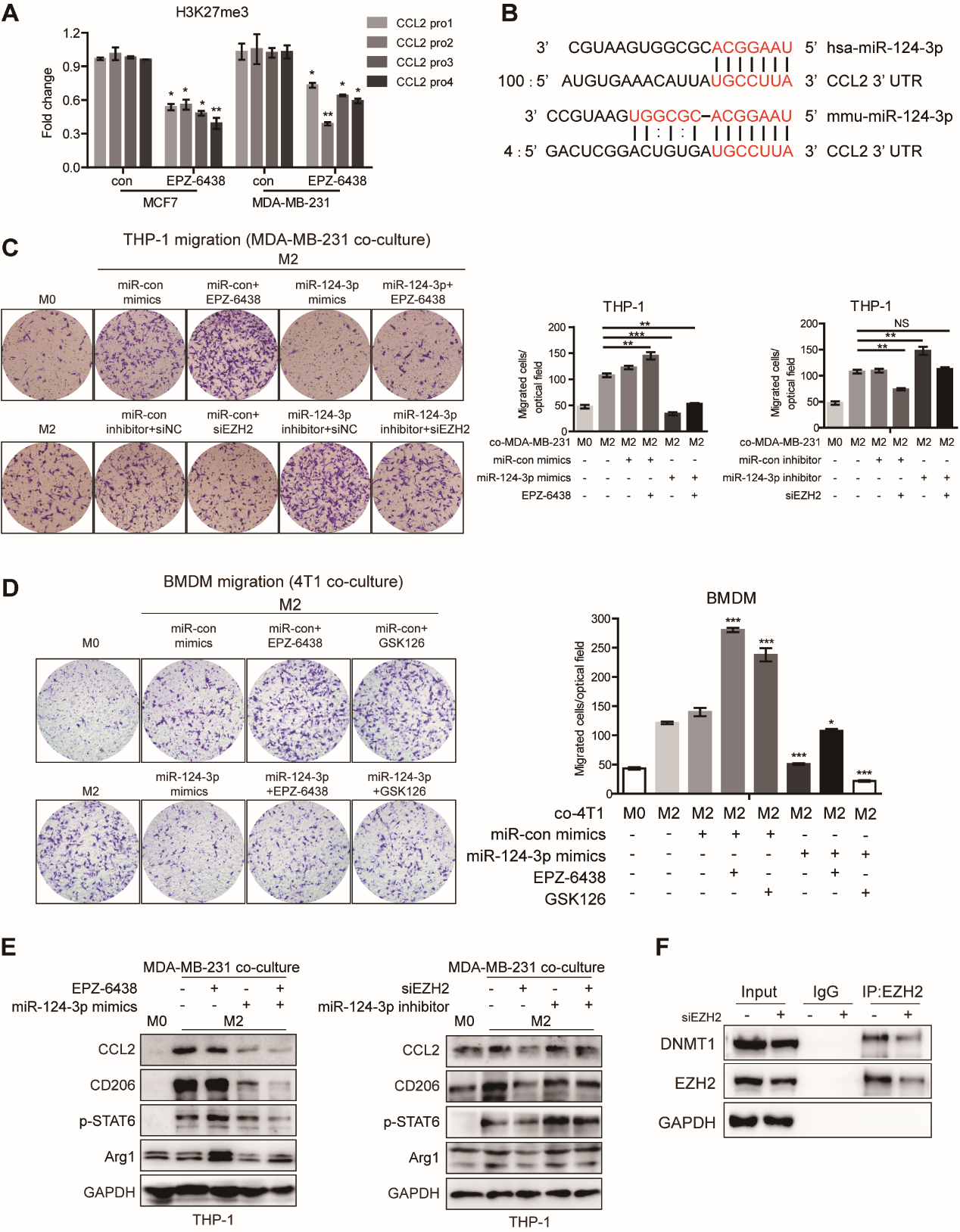


**Figure S5 related to Figure 4, miR-124-3p regulated by EZH2 manipulates CCL2 expression and macrophage polarization**

**(A)** CHIP-qPCR was performed to analyze the H3K27me3 level at the promoter of *Ccl2* after EPZ-6438 treatment. **(B)** MicroRNA.org Targets and Expression software predicted the miR-124-3p binding site in *Ccl2* 3’UTR region. **(C)** Transwell migration assays of THP-1-derived macrophage cells co-cultured with MDA-MB-231 cells carrying miR-124-3p mimics or inhibitors. Total number of cells in five fields was counted manually. **(D)** Transwell migration assays of BMDM M2 macrophages co-cultured with 4T1 treated with EZH2 inhibitors and/or miR-124-3p mimics for 48 hr. **(E)** THP-1-derived macrophage cells were co-cultured with MDA-MB-231 cells carrying miR-124-3p mimics treated with or without EPZ-6438, or carrying miR-124-3p inhibitors treated with or without siEZH2 for 48 hr. And M2 macrophage markers were analyzed by western blot. **(F)** Co-immunoprecipitation was conducted to test the direct binding between EZH2 and DNMT1 in MDA-MB-231 cells. In (**A, C** and **D**), all data were shown as mean values ± SD (n = 3). Statistical significance was addressed using unpaired, two‐tailed Student's t‐test, *p < 0.05, **p < 0.01, ***p < 0.001, NS, not significant. The statistical significance was calculated compared to the negative control (NC) in each group.

**Table S1. Key resources information**

| REAGENT or RESOURCE | SOURCE | IDENTIFIER |
| --- | --- | --- |
| Antibodies | | |
| H3K27me3 | Cell Signaling Technology | Cat# 9733 |
| Histone H3 | Proteintech Group | Cat# 17168-1-AP |
| EZH2 | Cell Signaling Technology | Cat# 5246 |
| β-actin | Proteintech Group | Cat# 66009-1-lg |
| GAPDH | Proteintech Group | Cat# 60004-1-lg |
| F4/80 | Abcam | Cat# ab240946 |
| CD163 | Abcam | Cat# ab182422 |
| CD206 | Abcam | Cat# ab125028 |
| TGF-β | Cell Signaling Technology | Cat# 3711 |
| STAT6 | Cell Signaling Technology | Cat# 5397 |
| Phospho-Stat6 (Tyr641) | Cell Signaling Technology | Cat# 56554 |
| STAT3 | Cell Signaling Technology | Cat# 9139 |
| Phospho-Stat3 (Tyr705) | Cell Signaling Technology | Cat# 9145 |
| CCL2 (mouse) | Abcam | Cat# ab25124 |
| CCL2 (human) | Abcam | Cat# ab 9669 |
| IL-10 | Cell Signaling Technology | Cat# 12163s |
| Arginase-1 | Cell Signaling Technology | Cat# 93668 |
| PPARγ | Cell Signaling Technology | Cat# 2435 |
| DNMT1 | Cell Signaling Technology | Cat# 5032 |
| Chemicals, Peptides, and Recombinant Proteins | | |
| EPZ-6438 | Selleck Chemicals | Cat# S7128 |
| GSK126 | MedChem Express | Cat# HY-13470 |
| Gefitinib | Selleck Chemicals | Cat# S1025 |
| RS 504393 | MedChem Express | Cat# HY-15418 |
| Bindarit | MedChem Express | Cat# HY-B0498 |
| BMS CCR2 22 | Tocris Bioscience | Cat# 3129 |
| DMSO | Sigma-Aldrich | Cat# BML-KI597-0400 |
| HEPES | Biosharp | Cat#: BS029B |
| DAPI | Invitrogen | Cat#: P36931 |
| PBS (phosphate buffer saline) | Beyotime Biotechnology | Cat#: C0221A |
| NP-40 | Beyotime Biotechnology | Cat#: ST366 |
| SDS | Biosharp | Cat#: BS028B |
| Protease inhibitor cocktail | Roche | Cat#: 04693159001 |
| Phosphatase inhibitor cocktail | Roche | Cat#: 04906837001 |
| Critical Commercial Assays | | |
| BCA Protein Assay Kit | ThermoFisher Scientific | Cat# 23227 |
| SimpleChIP® Plus Enzymatic Chromatin IP Kit (Magnetic Beads) | Cell Signaling Technology | Cat# 9005 |
| HiScript II 1st Strand cDNA Synthesis Kit (+gDNA wiper) | Vazyme | Cat# R223-01 |
| ChamQ SYBR qPCR Master Mix | Vazyme | Cat# Q311-02 |
| Human IL-10 ELISA Kit | Dakewe | Cat# 1111002 |
| Mouse IL-10 ELISA kit | Dakewe | Cat# DKW12-2100-096 |
| Mouse MCP-1 ELISA kit | Dakewe | Cat# DKW12-2739-096 |
| miRNeasy Mini Kit | QIAGEN | Cat# 217004 |
| QIAamp DNA Mini Kit | QIAGEN | Cat# 51304 |
| EpiTect Fast Bisulfite Kit | QIAGEN | Cat# 59824 |
| EpiScope® MSP Kit | TAKARA | Cat# R100B |
| Mir-X miRNA First-Strand Synthesis and TB Green RT-qPCR | TAKARA | Cat# 638314.0 |
| All-in-one miRNA RT-qPCR detection kit | genecopoeia | Cat# QP016 |
| Cultrex 3D Culture BME Cell Invasion Assay, 96 well | R&D systems | 3500-096-K |
| Recombinant DNA | | |
| pLenti-sgEZH2 E9.3 | Addgene | Cat# 90684 |
| pLenti-sgRNA | Addgene | Cat# 71409 |
| Software and Algorithms | | |
| ImageJ | Freeware | N/A |
| GraphPad Prism software 5.0 | N/A | https://www.graphpad.com/ |
| Other | | |
| Lipofectamine 2000 | ThermoFisher Scientific | Cat# 11668019 |
| Lipofectamine RNAiMAX | ThermoFisher Scientific | Cat# 13778150 |
| Goat anti-Mouse IgG (H+L) Cross-Adsorbed Secondary Antibody, Alexa Fluor 633 | ThermoFisher Scientific | Cat# A-21050 |
| Goat anti-Rabbit IgG (H+L) Secondary Antibody, Alexa Fluor 488 conjugate | ThermoFisher Scientific | Cat# A-11008 |
| Fetal bovine serum | Gibco | Cat# [10099-141](http://www.labbase.net/Product/ProductItems-27-188-1761-67972.html) |

**Table S2. The target sequences of siRNAs**

| **Name** | **Target sequences** |
| --- | --- |
| siEZH2 #2 | GCUGAAGCCUCAAUGUUUA |
| siEZH2 #3 | GAAUGGAAACAGCGAAGGA |
| siCCL2 #1 | GCUGUUAUAACUUCACCAATT |
| siCCL2 #2 | CUCGCGAGCUAUAGAAGAATT |
| has-mir-124-3p mimics | UAAGGCACGCGGUGAAUGCC |
| mmu-mir-124-3p mimics | UAAGGCACGCGGUGAAUGCC |
| hmu-mir-124-3p inhibitors | GGCAUUCACCGCGUGCCUUA |
| mmu-mir-124-3p inhibitors | GGCAUUCACCGCGUGCCUUA |
| LV3-sh hsaCCL2 | CTCGCGAGCTATAGAAGAA |
| LV3-sh musCCL2 | TCAGCACAGACCTCTCTCT |

**Table S3. Sequences of primers used in this study**

| **Sequences in qRT-PCR analysis** | | | |
| --- | --- | --- | --- |
| Genes | Forward (5’-3’) | | Reverse (5’-3’) |
| mus RPS18 | TCATGCAGAACCCACGACAGTACA | | TGTTGTCTAGACCGTTGGCCAGAA |
| mus CD206 | CATGAGGCTTCTCCTGCTTCT | | TTGCCGTCTGAACTGAGATGG |
| mus Arg1 | CTGGCAGTTGGAAGCATCTCT | | GTGAGCATCCACCCAAATGAC |
| mus Ym1 | ATCTATGCCTTTGCTGGAATGC | | TGAATGAATATCTGACGGTTCTGAG |
| mus Ccl2 | TTGGTGGGGTCTTTATCCGC | | GGCGCACTTCCTCCTGAAT |
| mus INOS | CAGCTGGGCTGTACAAACCTT | | CATTGGAAGTGAAGCGTTTCG |
| mus Il1b | AGTTGACGGACCCCAAAAG | | TTTGAAGCTGGATGCTCTCAT |
| mus TNFα | GCTCTTCTGTCTACTGAACTTCGG | | ATGATCTGAGTGTGAGGGTCTGG |
| hsa GAPDH | GAAGGTGAAGGTCGGAGT | | GAAGATGGTGATGGGATTTC |
| has RPS18 | ATTAAGGGTGTGGGCCGAAG | | GGTGATCACACGTTCCACCT |
| hsa Ccl2 | GTGTCCCAAAGAAGCTGTGATCT | | TGTCCAGGTGGTCCATGGA |
| has Arg1 | CAGATATGCAGGGAGTCACC | | CAGAAGAATGGAAGAGTCAG |
| hsa CD206 | GCTACATGGCGGTGGAGACAA | | ATGATGAGAGGCAGCAAGATGG |
| hsa-miR-124-3p | CGACGTAAGGCACGCG | | CAGTGCAGGGTCCGAGGTAT |
| mmu-miR-124-3p | TCTTTAAGGCACGCGGTG | | TATGGTTTTGACGACTGTGTGAT |
| hsa-U6 | CGAGCACAGAATCGCTTCA | | CTCGCTTCGGCAGCACATAT |
| mmu-U6 | CTCGCTTCGGCAGCACA | | AACGCTTCACGAATTTGCGT |
| **Sequences in MSP PCR assay** | | | |
| MSP-M (-1164–-997) | | TATCGAGTTGGTAGTGATTGTAGTC | CAATAAAATAAAAATTAAAAATCGTC |
| MSP-U (-1164–-997) | | TTGAGTTGGTAGTGATTGTAGTTGG | CAATAAAATAAAAATTAAAAATCATC |
| MSP-M (-972–-731) | | TATCGAGTTGGTAGTGATTGTAGTC | CAATAAAATAAAAATTAAAAATCGTC |
| MSP-U (-972–-731) | | TTGAGTTGGTAGTGATTGTAGTTGG | AATAAAATAAAAATTAAAAATCATC |
| MSP-M (-722–-605) | | TATCGAGTTGGTAGTGATTGTAGTC | CAATAAAATAAAAATTAAAAATCGTC |
| MSP-U (-722–-605) | | TTGAGTTGGTAGTGATTGTAGTTGG | ACAATAAAATAAAAATTAAAAATCATC |
