# Supplementary material for: Regulation of CCL2 by EZH2 affects tumor-associated macrophages polarization and infiltration in breast cancer
Source: Cell Death Dis. 2022 Aug 29;13(8):748. doi: 10.1038/s41419-022-05169-x (PMC9424193; doi:10.1038/s41419-022-05169-x)
Supplement: Supplementary file 2 — Supplemental Meterial [file 41419_2022_5169_MOESM2_ESM.pdf]

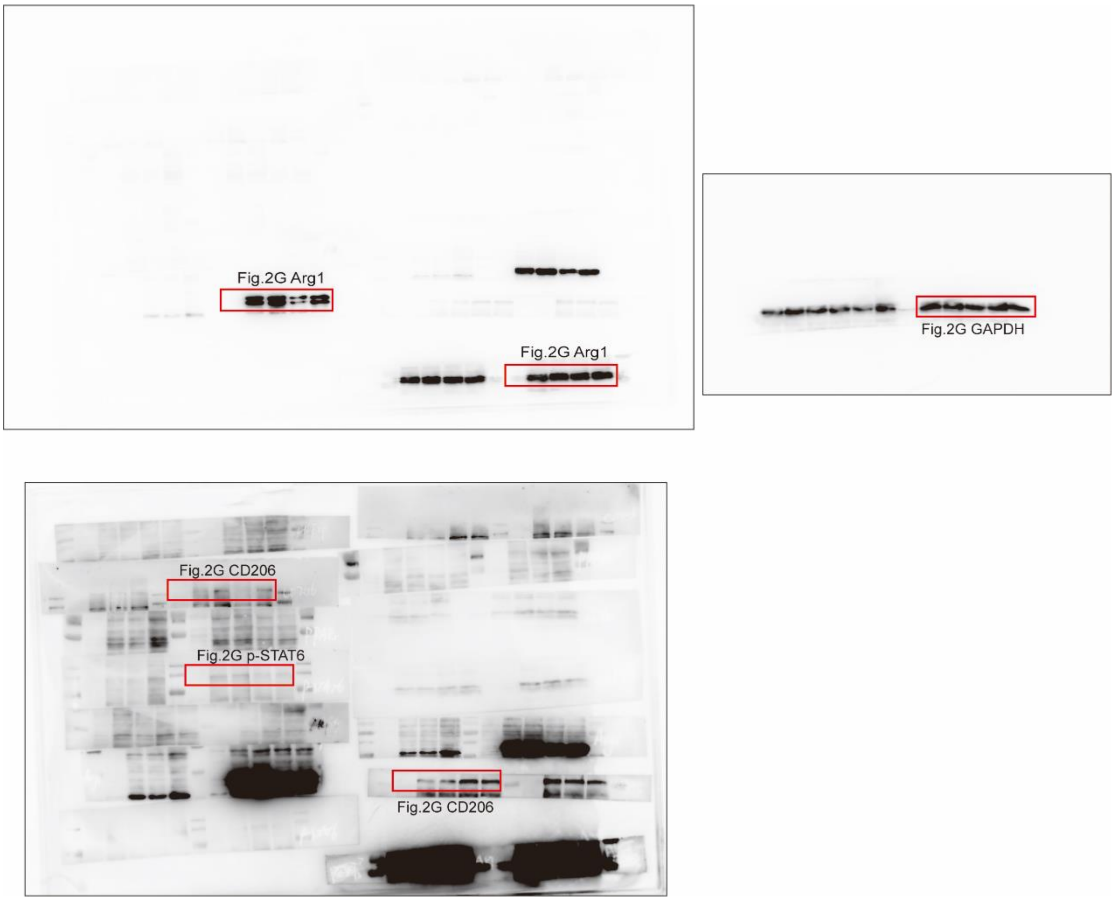

Supplemental Material 1. Full length uncropped original western blots in Figure 2

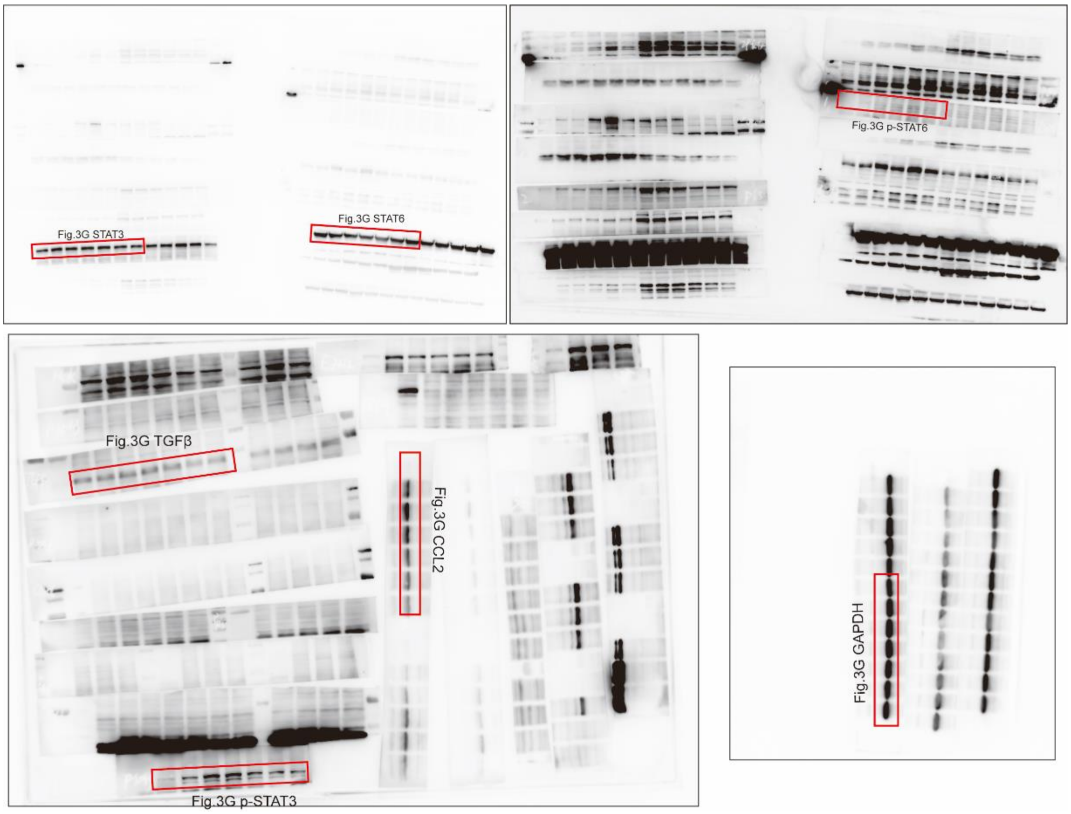

Supplemental Material 2. Full length uncropped original western blots in Figure 3

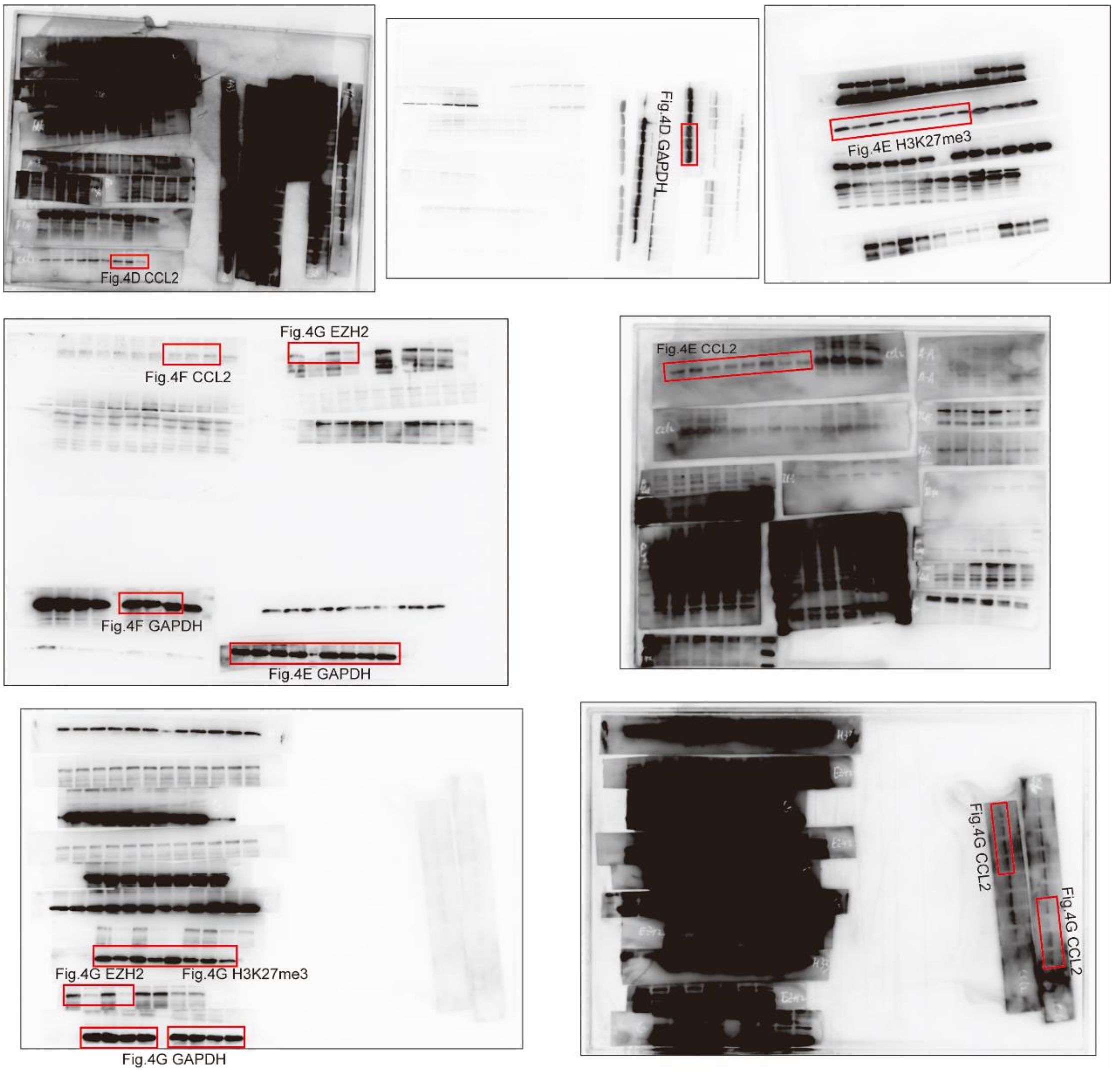

Supplemental Material 3. Full length uncropped original western blots in Figure 4

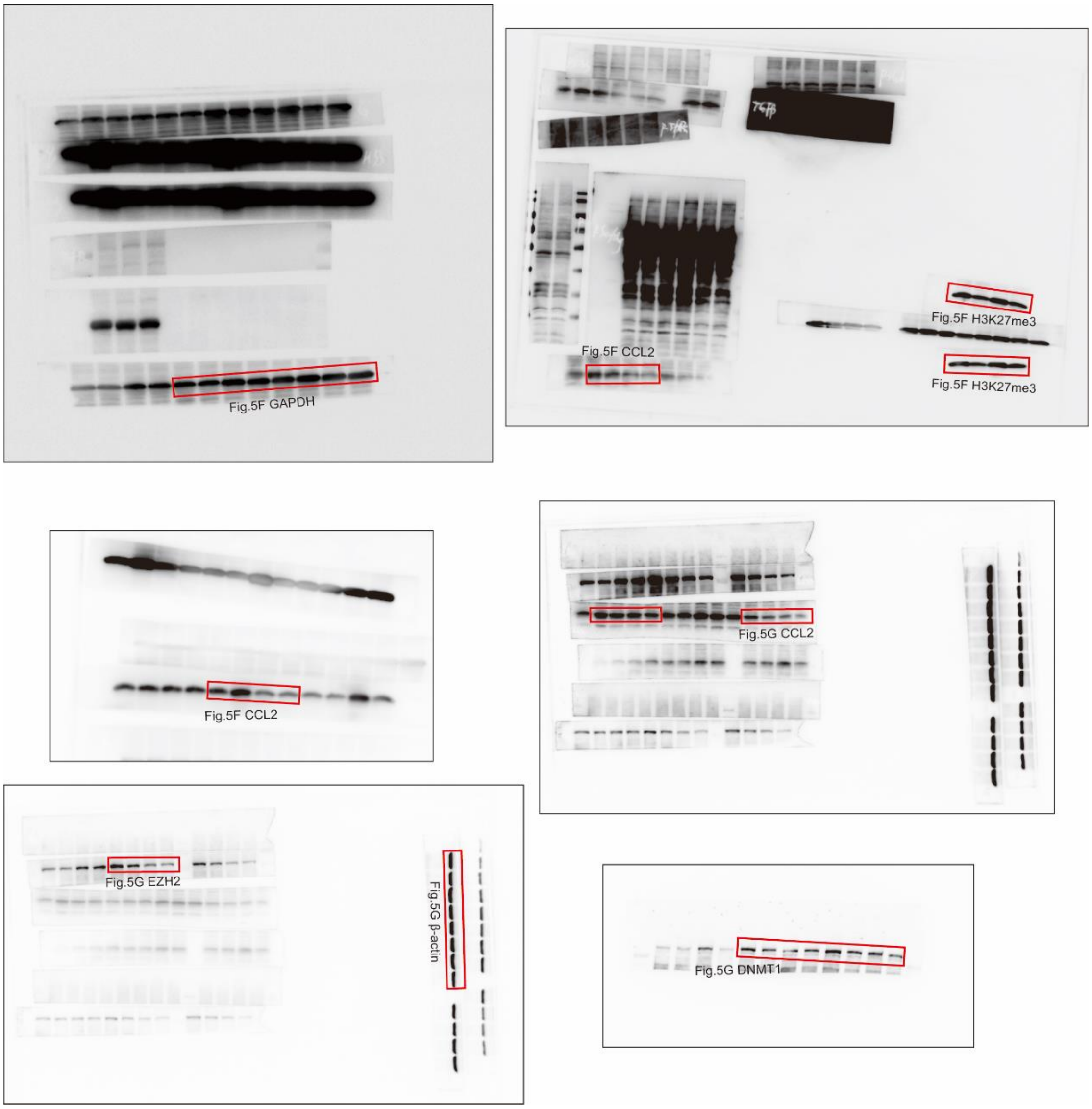

Supplemental Material 4. Full length uncropped original western blots in Figure 5

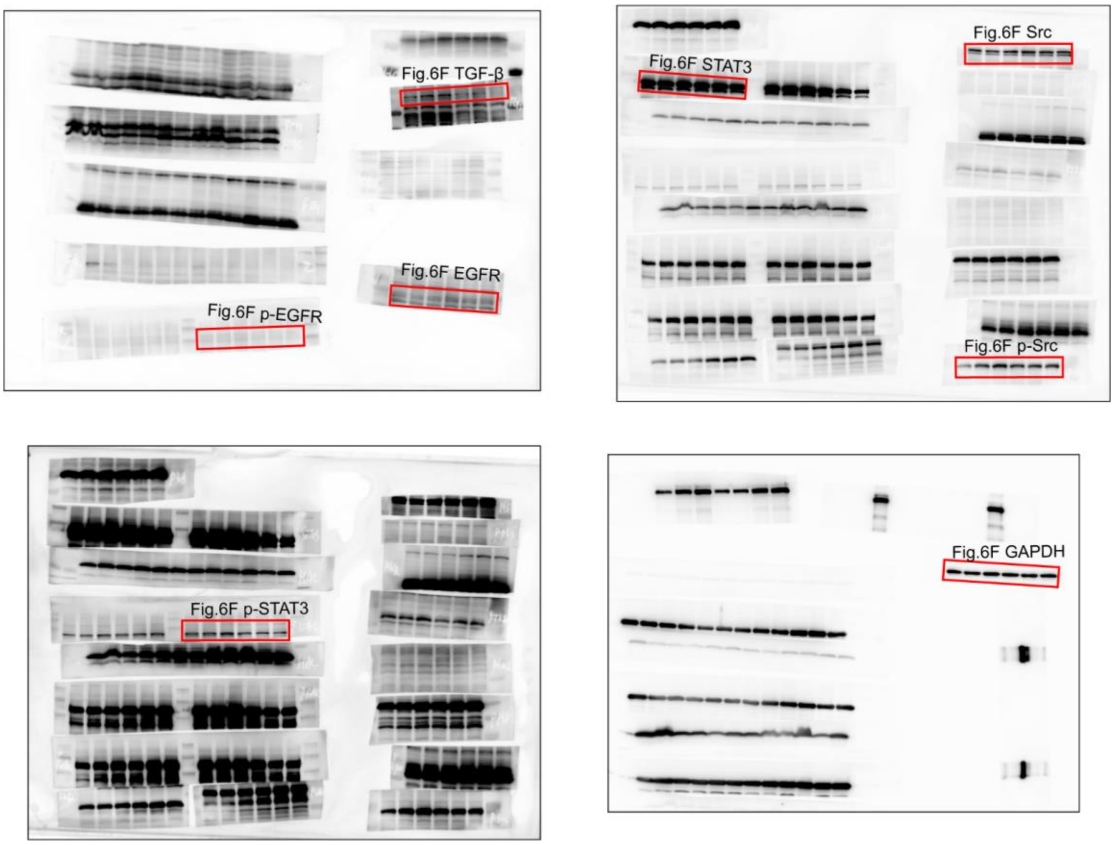

Supplemental Material 5. Full length uncropped original western blots in Figure 6
